# A Bioinspired Flexible Sensor for Electrochemical Probing of Dynamic Redox Disequilibrium in Cancer Cells

**DOI:** 10.1002/advs.202304079

**Published:** 2023-11-09

**Authors:** Zhongyuan Zeng, Jian Wang, Shuang Zhao, Yuchan Zhang, Jingchuan Fan, Hui Wu, Jiajia Chen, Zaikuan Zhang, Zexuan Meng, Lu Yang, Renzhi Wang, Bo Zhang, Guixue Wang, Chen‐Zhong Li, Guangchao Zang

**Affiliations:** ^1^ Institute of Life Science and Laboratory of Tissue and Cell Biology Lab Teaching & Management Center Chongqing Medical University Chongqing 400016 P. R. China; ^2^ Department of Pathophysiology Chongqing Medical University Chongqing 400016 P. R. China; ^3^ Key Laboratory for Biorheological Science and Technology of Ministry of Education State and Local Joint Engineering Laboratory for Vascular Implants Bioengineering College of Chongqing University Chongqing 400030 P. R. China; ^4^ Jinfeng Laboratory Chongqing 401329 P. R. China; ^5^ The M.O.E. Key Laboratory of Laboratory Medical Diagnostics The College of Laboratory Medicine Chongqing Medical University Chongqing 400016 P. R. China; ^6^ Bioelectronics and Biosensors Center School of Medicine Chinese University of Hong Kong Shenzhen 2001 Longxiang Avenue, Longgang District Shenzhen 518172 P. R. China

**Keywords:** ascorbic acid, hydrogen peroxide, ion‐exchange mechanisms, metal–organic frameworks, redox disequilibrium

## Abstract

Malignant tumors pose a serious risk to human health. Ascorbic acid (AA) has potential for tumor therapy; however, the mechanism underlying the ability of AA to selectively kill tumor cells remains unclear. AA can cause redox disequilibrium in tumor cells, resulting in the release of abundant reactive oxygen species, represented by hydrogen peroxide (H_2_O_2_). Therefore, the detection of H_2_O_2_ changes can provide insight into the selective killing mechanism of AA against tumor cells. In this work, inspired by the ion‐exchange mechanism in coral formation, a flexible H_2_O_2_ sensor (PtNFs/CoPi@CC) is constructed to monitor the dynamics of H_2_O_2_ in the cell microenvironment, which exhibits excellent sensitivity and spatiotemporal resolution. Moreover, the findings suggest that dehydroascorbic acid (DHA), the oxidation product of AA, is highly possible the substance that actually acts on tumor cells in AA therapy. Additionally, the intracellular redox disequilibrium and H_2_O_2_ release caused by DHA are positively correlated with the abundance and activity of glucose transporter 1 (GLUT1). In conclusion, this work has revealed the potential mechanism underlying the ability of AA to selectively kill tumor cells through the construction and use of PtNFs/CoPi@CC. The findings provide new insights into the clinical application of AA.

## Graphical abstract

A novel H_2_O_2_ assay platform (PtNFs/CoPi@CC) is constructed to monitor the redox disequilibrium in tumor cells induced by AA/DHA. The findings provide vital clues about the underlying mechanism of AA, suggesting that DHA, the oxidation product of AA, is highly possible the substance that actually acts on tumor cells in AA therapy.

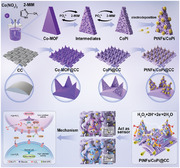

## 1 Introduction

The incidence of malignant tumors continues to rise each year,^[^ ^1^, ^2^ ^]^ posing a significant threat to human health and causing a significant global public health problem.^[^ ^2^ ^]^ Malignant tumor tissues can cause local structural and functional abnormalities in cell tissues and severe pain. The development of tumor tissues and the involvement of distant organs through hematogenous metastasis, lymphatic metastasis, and implantation metastasis cause multi‐system dysfunction, eventually leading to the death of patients.^[^ ^3^, ^4^ ^]^ Drug therapy is one of the primary therapeutic approaches to tumor treatment. At present, many chemotherapy drugs, such as methotrexate, cyclophosphamide, bleomycin, and cisplatin are used clinically; however, these therapeutic drugs often cause severe side effects, including hair loss, gastrointestinal reactions, bone marrow suppression, and other adverse reactions.^[^ ^5^, ^6^, ^7^, ^8^ ^]^ In addition to traditional chemical drugs, immune checkpoint inhibitors, such as the programmed death receptor 1 inhibitor—nivolumab (O‐drug, Opdivo) and the cytotoxic T lymphocyte‐associated antigen 4 inhibitors—ipilimumab (Y‐drug, Yervoy) are used for cancer treatment; however, their widespread application is limited by their high costs, making them unaffordable for many patients.^[^ ^9^ ^]^ Ascorbic acid (AA) is a promising therapeutic option for cancer treatment due to its low cost and minimal side effects.^[^ ^10^, ^11^, ^12^, ^13^ ^]^ Since Pauling and Cameron first used AA to treat cancer in 1971,^[^ ^14^ ^]^ several clinical studies have shown that AA can selectively kill tumor cells.^[^ ^15^, ^16^, ^17^, ^18^ ^]^ However, the mechanism underlying the ability of AA to selectively kill tumor cells remains unknown. Moreover, it is not clear why AA has different therapeutic effects on different types of tumors. Studies have shown that AA can cause oxidative stress‐mediated apoptosis in tumor cells,^[^ ^19^, ^20^ ^]^ accompanied by the release of a large number of reactive oxygen species (ROS), represented by hydrogen peroxide (H_2_O_2_); this may be closely related to redox disequilibrium in tumor cells.^[^ ^21^, ^22^ ^]^ Therefore, real‐time monitoring of the dynamics of H_2_O_2_ in tumor cells may provide insight into the potential molecular mechanism underlying the tumor cell‐killing ability of AA and may promote its clinical application in tumor therapy.

Electrochemistry has been extensively used to detect and monitor H_2_O_2_, due to its inherent advantages, including its fast response, low cost, real‐time detection, high sensitivity, and high selectivity.^[^ ^23^ ^]^ The work of many scholars has resulted in the development of a series of advanced flexible H_2_O_2_ sensors based on flexible substrates,^[^ ^24^ ^]^ such as polyimide‐laser‐engraved porous graphene,^[^ ^25^ ^]^ carbon fiber,^[^ ^26^ ^]^ and graphene fiber.^[^ ^27^ ^]^ However, detecting endogenous H_2_O_2_ in cells remains challenging due to the small content of H_2_O_2_ and its short half‐life.^[^ ^23^ ^]^ Moreover, the intracellular microenvironment is complex,^[^ ^28^ ^]^ containing many interfering substances, and cells are sensitive to mechanical forces. A promising approach to solve these problems is to combine the advantages of electrochemical sensors, such as their high sensitivity, specificity, and spatial and temporal resolution, with the use of flexible electrodes as substrates in order to minimize the distance between cells and the detection platform while also ensuring that the cells are not damaged by mechanical stress.^[^ ^29^, ^30^ ^]^ However, modifying the substrate to obtain more active sites while ensuring its flexibility in order to achieve sensitive detection of subtle intracellular chemical signals remains an important challenge. Metal–organic frameworks (MOFs) are new materials composed of metal nodes and organic connectors; their structure can be controlled by changing metal ions, organic bridging ligands, and reaction conditions.^[^ ^31^, ^32^ ^]^ Given their advantages, including their controllable structure and high specific surface area, they have broad application prospects in relation to gas storage,^[^ ^33^ ^]^ sensors,^[^ ^34^ ^]^ catalysts,^[^ ^35^, ^36^ ^]^ energy storage,^[^ ^37^ ^]^ and so on.^[^ ^31^ ^]^ Among them, nickel‐based MOF (Ni‐MOF) has low conductivity and limited exposure to active sites,^[^ ^38^ ^]^ copper‐based MOF (Cu‐MOF) is restricted by their saturated coordination with few active sites,^[^ ^39^ ^]^ and iron‐based MOFs (Fe‐MOFs) face various challenges, such as a lack of detection sensitivity and selectivity when applied to sensing.^[^ ^40^ ^]^ In contrast, cobalt‐based MOF (Co‐MOF) has the characteristics of growing uniformly in different interfaces, does not require the use of a binder, which occupies the catalytic active center and hinders the transmission of electrons or electrolyte ions, and does not require material diffusion during modification.^[^ ^41^ ^]^ The method of in situ growth on a flexible substrate is the best choice for modifying electrodes to detect cell‐derived H_2_O_2_. However, the poor conductivity of Co‐MOF itself and the structural instability of organic ligand 2‐methylimidazole (2‐MIM) caused by protonation in acidic conditions and deprotonation in alkaline conditions limit its application for biomolecule detection and analysis.^[^ ^32^, ^42^, ^43^ ^]^ At present, many studies have used Co‐MOF as a precursor and sacrificial template and have modified it to increase its stability and conductivity. For example, mesoporous Co_3_O_4_ nanowires were synthesized by solid‐phase pyrolysis;^[^ ^44^ ^]^ Co–N–C flakes were prepared by high‐temperature carbonization;^[^ ^35^ ^]^ and other cobalt‐containing oxides,^[^ ^45^ ^]^ sulfides, and layered double hydroxides were prepared by high‐temperature oxidation, sulfidation, and the hydrothermal synthetic procedure, respectively.^[^ ^46^, ^47^ ^]^ However, there a several disadvantages of the above, including the need for many complicated procedures and the requirement for high‐temperature, high‐pressure environments. This limits the safe, comprehensive, and wide application of these modification schemes.

In this work, inspired by the ion exchange process during coral formation which improves the stability of the materials,^[^ ^48^, ^49^ ^]^ an energy‐saving, safe, simple, and feasible strategy for preparing porous cobalt phosphate (CoPi) nanoarrays was proposed. The approach involved the use of the ion exchange method in a phosphate solution to enhance the conductivity and stability of Co‐MOF. Subsequently, platinum nanoflowers (PtNFs) were grown in situ on a coral‐like microarray electrode via electrodeposition to improve the detection sensitivity of the sensor. Finally, a novel real‐time in situ detection platform (PtNFs/CoPi@CC) for intracellular H_2_O_2_ was constructed. The platform exhibited excellent sensitivity, anti‐interference, stability, and reproducibility, among other performance metrics, substantially exceeding the level of other similar electrodes. This provided a foundation to further explore the molecular mechanism of AA in the treatment of tumors. To this end, the current results revealed that dehydroascorbic acid (DHA), the oxidation product of AA, induced the release of H_2_O_2_ during dynamic redox disequilibrium in cancer cells. This release was positively correlated with the number and activity of glucose transporter 1 (GLUT1) on the cell membrane. These findings provide new insight into the mechanism underlying the selective killing of tumor cells by AA and its differential clinical efficacy. In the future, this novel real‐time in situ detection platform may have great application potential for the study of intracellular signaling pathways and the development of new potential therapeutic strategies.

## 2 Results and Discussion

### 2.1 Design and Construction of the PtNFs/CoPi@CC Electrochemical Sensing Platform

**Figure 1:**
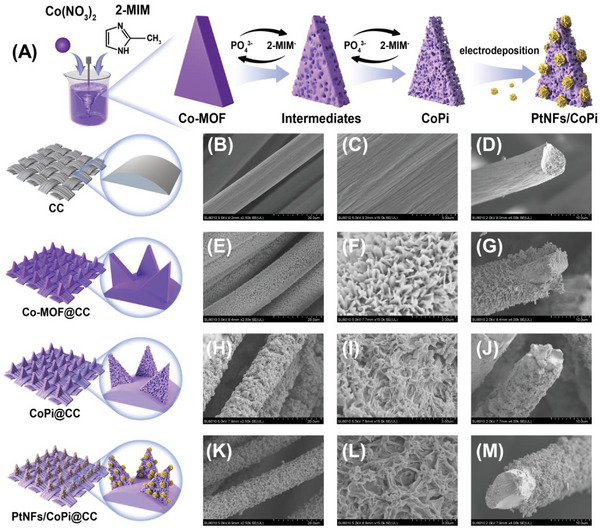
The construction process and physical characterization of the PtNFs/CoPi@CC. A) Formation mechanism of Co‐MOF derived PtNFs/CoPi composite based on ion exchange reaction. SEM images of electrodes: B) bare CC 20 µm and C) 3 µm and D) cross‐sectional image, Co‐MOF@CC E) 20 µm and F) 3 µm and G) cross‐sectional image, CoPi@CC H) 20 µm and I) 3 µm and J) cross‐sectional image, and PtNFs/CoPi@CC K) 20 µm and L) 3 µm and M) cross‐sectional image.

**Figure** **1** shows the preparation of PtNFs/CoPi@CC as observed by scanning electron microscope (SEM). The flexible sensing platform (Figure 1A) was constructed by in situ growth of PtNFs/CoPi materials on the surface of smooth carbon cloth (CC) via room temperature crystallization, etching, and electrodeposition in sequence (see Figure 1B‐D; Figure S1A, Supporting Information). First, Co‐MOF with 2D blade structures was grown on the surface of the CC using the room temperature crystallization method (Figure 1E‐G; Figure S1B, Supporting Information). Second, inspired by the ion exchange reaction that occurs during the formation of coral in seawater to form porous, stable structure, we proposed the synthesis of stable MOF derivative CoPi utilizing the ion exchange reaction in phosphate solution at room temperature via the one‐step etching method. Given that the coordination bonds between metal nodes and organic ligands in MOF are weak and reversible, these bonds in the skeleton can be easily broken; thus, ion exchange reactions can occur at room temperature. Accordingly, the deprotonated 2‐methlymidazole (2‐MIM^−^) ligand in Co‐MOF was exchanged with PO_4_^3−^ in phosphate buffer solution (PBS). Then, 2‐MIM^−^ was uniformly removed by in situ corrosion, leaving the metallic portion to coordinate with PO_4_^3−^ in solution in order to obtain coral‐like CoPi (more details on the synthesis reaction mechanism of Co‐MOF and CoPi are provided in Notes S1 and S2, Supporting Information). The etching time is an important factor in the occurrence of ion exchange reactions. After etching for 12 h, some sheet‐like MOF structures were still on the surface of the carbon fibers (Figure S2, Supporting Information), indicating that the ion exchange reaction was not complete. However, after 24 h of etching, the coral‐like array structure was stable with no residual leaf‐like MOF structure (Figure 1H,I; Figure S1C, Supporting Information). The cross‐sectional view (Figure 1J) of CoPi@CC also shows that the coral‐like array structure was uniformly distributed on the carbon fibers. This unique electrode design has several advantages: First, the ion exchange (etching) process occurred at the two‐phase interface. By using this method, the structure and size of the original MOF were well preserved, and the unique bladed structure was transformed into a porous coral‐like structure. This unique structure allowed the active material to be effectively exposed to participate in electrochemical reactions and rapid electron transfer. Second, a flexible conductive CC closely connected to the CoPi array can be used directly as an electrode without any binder additive, ensuring high mechanical stability and electrical conductivity. Third, the unique porous coral‐like structure of CoPi had a high specific surface area, providing more active sites for catalysis, which is conducive to improved electrochemical performance. Fourth, the whole preparation process was carried out at room temperature, which is energy‐saving and efficient. Finally, to further improve the detection performance of the sensor, PtNFs were grown by in situ electrodeposition on the coral‐like porous surface of CoPi, which provided more growth sites for the growth of PtNFs. The results in Figure 1K–M and Figure S1D (Supporting Information) show that many PtNFs were evenly distributed in the CoPi layer. Therefore, the structural characteristics of the PtNFs/CoPi@CC sensing platform, such as its porosity, stability, and the growth of many PtNFs, are conducive to the adhesion of living cells, provide a shorter distance between the catalytic materials and H_2_O_2_ released by cells, and reduce the loss of H_2_O_2_, thereby providing excellent support for the in situ real‐time detection of intracellular H_2_O_2_.

### 2.2 Characterization of PtNFs/CoPi@CC

**Figure 2:**
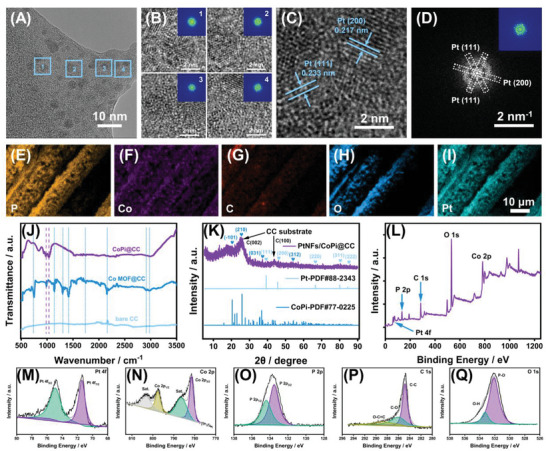
Physical characterization of the PtNFs/CoPi@CC. A–D) TEM analysis. A) HRTEM image of PtNFs/CoPi@CC and B) HRTEM image of four selected different areas (blue square) of PtNFs/CoPi@CC. Insets in (B) are the corresponding color fast Fourier transform (FFT) pattern from different areas. C) Analysis of HRTEM image of PtNFs/CoPi@CC and D) the corresponding FFT pattern of the (C). Inset in (D) is the color pattern of (D). E–I) EDS element (P, Co, C, O, and Pt) mapping of PtNFs/CoPi@CC. Scale bar, 10 µm. J) FT‐IR spectra of bare CC, Co‐MOF@CC, and CoPi@CC. K) XRD spectrum of Pt, CoPi, and PtNFs/CoPi@CC. L) XPS wide‐scan survey spectra of PtNFs/CoPi@CC. High‐resolution XPS spectra of PtNFs/CoPi@CC: M) Co 2*p* spectrum, N) Pt 4*f* spectrum, O) O 1*s* spectrum, P) C 1*s* spectrum, and Q) P 2*p* spectrum.

The image obtained by transmission electron microscopy (**Figure** **2**A–D; Figures S3 and S4, Supporting Information) depicts the morphology and crystal structure of PtNFs/CoPi@CC, which are consistent with the results of SEM in Figure 1. The lattice spacings of 0.217 and 0.233 nm can be attributed to the (200) and (110) crystal planes of Pt, respectively. These results demonstrate that the dominant crystal planes formed by electrodeposition are Pt (200) and Pt (110). Previous studies have shown that the Pt atoms in the lattice of the Pt (111) surface change 9–24 times more than that of the Pt (200) surface during the decomposition of H_2_O_2_ catalyzed by Pt.^[^ ^50^ ^]^ Therefore, the highly sensitive catalytic performance of this sensor for H_2_O_2_ may be because H_2_O_2_ was adsorbed on its surface after first being captured by Pt (111) facets,^[^ ^51^ ^]^ and then the Pt (200) facets, whose lattice changes were much smaller, resulting in a more stable structure,^[^ ^50^ ^]^ provided an efficient catalytic effect on H_2_O_2_. In addition, elemental atlas analysis (EDS, Figure 2E–I; Figure S5, Supporting Information) confirmed the uniform distribution of P, Co, C, O, and Pt on the electrode. The chemical structure of PtNFs/CoPi@CC was characterized by its infrared diffraction pattern, as shown in Figure 2J. The absorption peaks in the wavelength ranging from 500–3000 correspond to the characteristic peaks of Co‐MOF (756, 1140, 1300, 1420, 1780, 2160, 2926, and 2955).^[^ ^52^ ^]^ The characteristic peaks of CoPi are 977 and 1035 cm^−1^.^[^ ^53^ ^]^ These data strongly confirm the successful preparation of PtNFs/CoPi composites on the CC surface. The X‐ray diffraction spectrum of PtNFs/CoPi@CC is shown in Figure 2K. The two distinct diffraction peaks at 25.6° and 43.4° correspond to the (002) and (100) crystal planes of the CC, respectively.^[^ ^54^, ^55^ ^]^ The five weak diffraction peaks at 2θ values of 39.3°, 45.7°, 66.6°, 80.1°, and 84.5° correspond to the (111), (200), (220), (311), and (222) crystal planes of Pt, respectively (PDF#88‐2343). The remaining four weak diffraction peaks, at 2θ values of 20.4°, 25.9°, 36.8°, and 54.4° match the (−101), (210), (031), and (312) crystal planes of CoPi (PDF#77‐0225),^[^ ^53^ ^]^ respectively. In addition, the chemical composition of PtNFs/CoPi@CC was investigated by complete X‐ray photoelectron spectroscopy (Figure 2L–Q). The characteristic peaks of spin orbits for P, Co, O, C, and Pt were consistent with those previously reported.^[^ ^53^, ^56^ ^]^ The above results indicate that the PtNFs/CoPi@CC electrode was successfully constructed.

We further elaborated on the details of the CoPi synthesis process from the perspectives of ion strength, ion volume, and coordination methods. According to the ion strength formula *I*_i_ $=\frac{1}{2}\sum_{i\;=\;1}^{n}\;$ *C*_i_ *Z*_i_^2^, due to its large coefficient of phosphate radical, it is the main factor affecting ion strength, the ion strength increases as the ion exchange reaction progresses (more details of the calculation of ion strength are shown in Notes S1 and S2, Supporting Information). Moreover, as the ion strength increases, the solubility of insoluble substances in the reaction system gradually decreases, making the obtained CoPi products more stable in the reaction system.^[^ ^57^ ^]^ The volume of phosphate radical is 159.92 × 10^−30^ m^3^ with a length of 5.626, a width of 5.313, and a height of 5.350 Å. The volume of deprotonated 2‐MIM is 162.61 × 10^−30^ m^3^ with a length of 7.259, a width of 5.384, and a height of 4.163 Å (Figure S6, Supporting Information). Therefore, we speculate that when the weak and reversible coordination bond between the metal node and the organic ligand in Co‐MOF breaks, the phosphate radical will occupy the vacancy of the deprotonated 2‐MIM due to its smaller volume than the deprotonated 2‐MIM, and then Co^2+^ and phosphate radical will form CoPi with a more stable ionization bond. The comparison in size also indicates the advantage of the reaction occurring in the direction of conversion to CoPi. Therefore, the increase of ion strength and the smaller volume of phosphate radical both promote the forward of ion exchange reactions. Based on our research on crystal structure, we found that in the crystal structure of Co‐MOF, Co^2+^ forms a 1:2 coordination bond with the deprotonated 2‐MIM.^[^ ^43^, ^58^ ^]^ At the beginning of the reaction, the coordination bond between Co^2+^ and the N atom which lost the proton in the deprotonated 2‐MIM breaks, subsequently, phosphate radical attracts closer to Co^2+^ through anisotropic charges and forms more stable ion bonds, the coordination between Co^2+^ and PO_4_^3−^ in Co_3_(PO_4_)_2_ is relatively complex, Co^2+^ occupy two distinct coordination polyhedra, one five and one six‐coordinated, in a ratio of two to one (Figure S7, Supporting Information).^[^ ^59^, ^60^, ^61^ ^]^

To obtain the best detection performance, the influence of the electrodeposition time of PtNFs on the detection performance of the sensor was first analyzed. The analysis of the H_2_O_2_ detection capability of the sensor obtained after electrodeposition from 1200 to 4800 s indicated that the longer the electrodeposition time, the higher the current response obtained during the detection of H_2_O_2_. However, when the electrodeposition time reached 6000 s, the current response generated by the sensor was considerably weakened. Therefore, 4800 s was finally selected as the electrodeposition time to construct the sensor (Figure S8A,B, Supporting Information). The influence of the working potential on the detection performance was further analyzed. The operating potential was set from −0.1 to −0.6 V and the current response for detecting H_2_O_2_ was examined. Figure S8C,D (Supporting Information) shows that the current response gradually increased from −0.1 to −0.5 V and then weakened when it increased to −0.6 V. Therefore, the optimal reduction current signal could be obtained when −0.5 V was used as the working potential. Thus, based on the above optimization, 4800 was ultimately selected as the electrodeposition time to construct the sensor, and the subsequent experiments were conducted at the working potential of −0.5 V.

**Figure 3:**
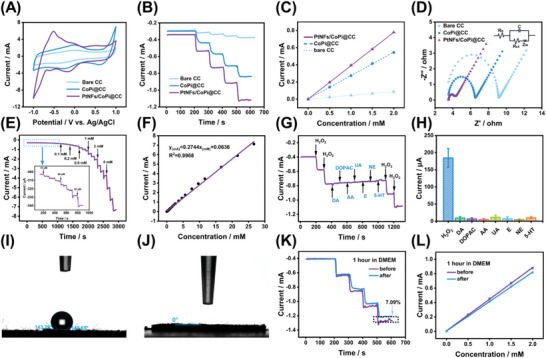
Electrochemical characterization of the PtNFs/CoPi@CC. A) CVs of the bare CC, CoPi@CC, and PtNFs/CoPi@CC in 0.1 m PBS solution (pH 7.40) containing 0 and 2 mm H_2_O_2_. Amperometric response of different electrodes (bare CC, CoPi@CC, and PtNFs/CoPi@CC) with successive additions of B) 0.5 mm H_2_O_2_ and C) the corresponding fitting line graph. D) Electrochemical Impedance Spectroscopy (EIS) obtained from different modified electrodes: bare CC, CoPi@CC, and PtNFs/CoPi@CC in 5 mm [Fe(CN)_6_]^3−/4−^ solution containing 0.1 m KCl. Amperometric response of PtNFs/CoPi@CC in 0.1 m PBS (pH 7.40) at −0.5 V upon successive additions of E) H_2_O_2_ from 10 µm to 5 mm (triple injections per concentration) and F) the corresponding calibration curves. Amperometric responses of G) the PtNFs/CoPi@CC to 100 µm dopamine (DA), 3,4‐dihydroxyphenylacetic acid (DOPAC), AA, uric acid (UA), adrenaline (E), norepinephrineNE, 5‐hydroxytryptam5‐HT, and 500 µm H_2_O_2_, respectively, in 0.1 m PBS and H) the corresponding statistical bar chart (*n* = 3). Typical static water contact angle of I) bare CC and J) PtNFs/CoPi@CC. K) Amperometric current responses obtained at the PtNFs/CoPi@CC upon successive additions of H_2_O_2_ (each addition, 0.5 mm) in 0.1 m PBS before and after the immersion in the culture medium for 1 h of PtNFs/CoPi@CC. L) Pre‐ and post‐calibration curves obtained from the PtNFs/CoPi@CC under the condition of (K).

To study the electrochemical activity of different electrodes for the reduction of H_2_O_2_, cyclic voltammograms (CVs) were measured in PBS containing 0 and 2 mm H_2_O_2_. **Figure** **3**A shows that the reduction currents of H_2_O_2_ on the PtNFs/CoPi@CC were ≈2.75 and 9.36 times higher than those on the CoPi@CC and bare CC, respectively. The excellent electrochemical performance of our developed sensor might be attributed to the coral‐like porous structure on the electrode surface, which facilitates fast mass transfer in the electrochemical process. The electrocatalytic capacities of the bare CC, CoPi@CC, and PtNFs/CoPi@CC sensor electrodes to H_2_O_2_ were studied using amperometry (*i–t*). Figure 3B,C demonstrates that with the continuous addition of H_2_O_2_, the current signals of the bare CC, CoPi@CC, and PtNFs/CoPi@CC electrodes all exhibited stepped responses, and the reduction current signal of the PtNFs/CoPi@CC electrode was remarkably greater than that of the CoPi@CC electrode and bare CC electrode. This indicates that the catalytic performance of the developed electrode to H_2_O_2_ was considerably improved with the layer‐by‐layer modification of catalytic materials. After adding different concentrations of H_2_O_2_ (0, 2, 4, 5, 6, and 8 mm) into the detection system (0.1 m PBS, pH 7.40), the CV response of PtNFs/CoPi@CC in the presence of different concentrations of H_2_O_2_ was determined, as shown in Figure S9 (Supporting Information). The CV curves exhibited good linearity at the reduction potential. These findings indicate that the reduction current was more sensitive to the detection of H_2_O_2_.

The charge‐transfer resistance of the bare CC, CoPi@CC, and PtNFs/CoPi@CC electrodes was studied by Electrochemical Impedance Spectroscopy (EIS) to evaluate the conductivity of the electrodes. Figure 3D shows that the impedance of bare CC, CoPi@CC, and PtNFs/CoPi@CC were 5.802, 2.966, and 0.647 Ω, respectively. The impedance value (*R*_CT_) of the electrode decreased gradually, indicating that PtNFs/CoPi@CC was prepared successfully and had good electrical conductivity. This result is mainly due to the coral‐like structure of the MOF‐derived CoPi, which provides many sites for the loading of PtNFs, accelerates electron transport, and thus, improves the electrical conductivity of the electrode. At the same time, the variation trend in the impedance value was consistent with the CV curve. This further demonstrates that the modification scheme can improve the conductivity of the electrode (Figure S10, Supporting Information). In addition, the CV obtained at different scan speeds in a solution containing 2 mm H_2_O_2_ showed that the peak current was proportional to the square root of the scan speed, indicating that the catalysis of H_2_O_2_ on the electrode surface was controlled by diffusion (Figure S11, Supporting Information).

Figure 3E shows the *i–t* curve obtained on PtNFs/CoPi@CC under optimal conditions. When the concentration of H_2_O_2_ was continuously increased in the 10 mL 0.1 m PBS solution, the catalytic current quickly rose to a stable value. The inset shows an *i–t* curve for the low concentration region (10–90 µm), indicating that the electrode could detect H_2_O_2_ at concentrations as low as 10 µm through the response of reduction currents. Figure 3F shows that PtNFs/CoPi@CC had a good linear relationship with H_2_O_2_ in the concentration range from 10 µm to 26.64 mm. The corresponding equation was I (mA) = 0.2744*C*_H2O2_ (mm)+0.0636 (*R* ^2^ = 0.9968), and the low limit of detection was calculated as 0.222 µm (S/N = 3). The detection limit of the proposed PtNFs/CoPi@CC sensor is comparable with that of previously reported sensors for the determination of H_2_O_2_, and the linear range exceeds those of all previously reported H_2_O_2_ sensors of the same type (Table S1, Supporting Information). PtNFs/CoPi@CC showed good selectivity against common interfering substances (Figure 3G,H; Figure S12, Supporting Information). The highly sensitive differential pulse voltammetry (DPV) results showed that the oxidation potential of these interfering substances was positive, whereas the reduction potential of H_2_O_2_ was negative. The huge difference in redox potential may be an important reason underlying the good selectivity of the electrode. In addition, the current recorded by PtNFs/CoPi@CC was more stable than that recorded by Co‐MOF@CC (Figure S13, Supporting Information), indicating that the developed electrode has excellent stability in recording current responses over long periods of time.

To investigate the antifouling property of the electrode, the water contact angle of PtNFs/CoPi@CC was first measured to evaluate the hydrophilicity of the electrode surface. Hydration forces are the key factor in determining whether a surface will reduce protein adsorption.^[^ ^62^, ^63^, ^64^ ^]^ Figure 3I,J shows that the static water contact angle on the surface of PtNFs/CoPi@CC was 0° (*n* = 3), which is much smaller than that of the bare CC (140.65°–143.29°, *n* = 3). The process of measurement is shown in Videos S1 and S2 (Supporting Information). The superhydrophilicity of PtNFs/CoPi@CC can be attributed to the increased carboxyl and hydroxyl groups on the surface of the CC by the treatment of the acid solution, as well as the water‐absorbing quality provided by the unique porous coral‐like structure of CoPi. In addition, electrodes were immersed in complete medium containing serum for 1 h and their amperometric responses to H_2_O_2_ were assessed. The results revealed that the current response of the developed electrode after immersion was 92.91% of that before immersion (Figure 3K,L). This indicates that the electrode has a good antifouling property, which guarantees the reliable detection of the electrode in a complex environment. Finally, the reproducibility and stability of the sensor were analyzed. The results showed that the five groups of sensors prepared in different batches exhibited good consistency in the detection of H_2_O_2_ (RSD = 2.28%). This indicates that the sensors have good reproducibility (Figure S14A, Supporting Information). The same group of sensors was prepared and placed at room temperature for 3, 6, 9, and 12 days to test their current responses to H_2_O_2_. Figure S14B (Supporting Information) shows that after 12 days, the developed sensor maintained 93.3% of its initial current response. This demonstrates that long‐term storage does not substantially affect its electrochemical performance.

### 2.3 Mechanism Underlying the Cancer Cell Killing Ability of AA

To verify the feasibility of the developed electrode for monitoring the release of H_2_O_2_ from living cells, the biocompatibility of the material was verified. First, the biocompatibility of the electrode was investigated by living/dead cell staining experiments. Figure S15A (Supporting Information) shows that PC12 cells grew well on the bare CC and PtNFs/CoPi@CC electrodes and maintained high vitality. Subsequently, the cytotoxicity of the PtNFs/CoPi material was determined by a CCK‐8 assay. Figure S15B (Supporting Information) shows that compared with bare CC, PtNFs/CoPi‐modified CC exhibited no obvious toxic effect on PC12 cells. Therefore, the constructed PtNFs/CoPi@CC sensor has excellent potential for the detection of living cells.

**Figure 4:**
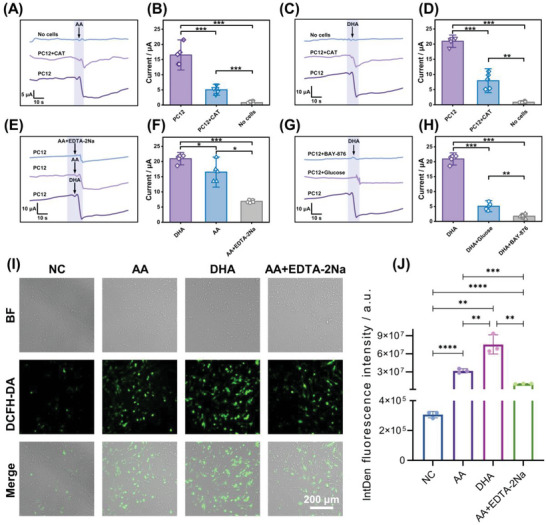
The mechanism of AA killing cancer cells. The current response of PtNFs/CoPi@CC to the addition of 500 µm AA to promote H_2_O_2_ release in the PBS containing no cells (blank group), PBS containing PC12 cells, as well as A) PBS containing PC12 cells and catalase (CAT) and B) the statistical analysis of corresponding current responses, *n* = 5. The current response of PtNFs/CoPi@CC to the addition of 500 µm DHA to promote H_2_O_2_ release in the PBS containing no cells (blank group), PBS containing PC12 cells, as well as C) PBS containing PC12 cells and CAT and D) the statistical analysis of corresponding current responses, *n* = 5. The current response of PtNFs/CoPi@CC to the addition of 500 µm AA or 500 µm DHA or 500 µm AA containing 100 µm EDTA‐2Na to promote H_2_O_2_ release in E) the PBS containing PC12 cells, respectively, and F) the statistical analysis of corresponding current responses, *n* = 5. The current response of PtNFs/CoPi@CC to the addition of 500 µm DHA to promote H_2_O_2_ release in the PBS containing PC12 cells, PBS containing PC12 cells and 27.5 mm glucose, as well as G) PBS containing PC12 cells and 100 µm BAY‐876 and H) the corresponding current responses, *n* = 5. Fluorescence imaging of I) the ROS levels in PC12 cells with different treatments and J) Quantitative analyses of ROS production, *n* = 3. Data are shown as mean values ± 1.5 standard deviations. Two‐tailed *t*‐test was used for statistical analysis (^*^ *p* < 0.05, ^**^ *p* < 0.01, or ^***^ *p* < 0.001).

Previous studies have indicated that AA may induce tumor cell apoptosis by inducing ROS release in cells, resulting in the selective killing of tumor cells.^[^ ^22^, ^65^ ^]^ Therefore, the constructed detection platform (PtNFs/CoPi@CC) was first applied to determine whether AA causes the release of H_2_O_2_ by tumor cells. **Figure** **4**A,B shows that the reduction current signal detected by the sensor was remarkably enhanced after AA stimulation, whereas the reduction current signal was substantially weakened when CAT was present in the electrolyte. This could be attributed to the specific decomposition of H_2_O_2_ by catalase. The substance released by AA‐induced PC12 cells was indeed H_2_O_2_. According to the linear curve obtained by the electrode in a complete culture medium containing fetal bovine serum (Figure S16, Supporting Information), the total H_2_O_2_ concentration produced by the tested cells were 70.21, 26.92, and 10.93 µm corresponding, consistent with reports in the literature at the same level of magnitude, although with slight differences in numerical values.^[^ ^66^, ^67^, ^68^, ^69^ ^]^

One study suggested that DHA, the oxidative product of AA, can induce the release of H_2_O_2_ in cancer cells, leading to oxidative stress‐mediated apoptosis.^[^ ^70^ ^]^ Therefore, the effect of DHA on H_2_O_2_ released from tumor cells was analyzed. Figure 4C,D reveals that DHA also induced the release of H_2_O_2_ from PC12 cells, the total H_2_O_2_ concentration produced by the tested cells were 86.88, 37.86, and 11.16 µm corresponding. Importantly, the concentration of H_2_O_2_ released by DHA stimulation was considerably higher than that released by AA stimulation (Figure 4E,F), the total H_2_O_2_ concentrations produced by the tested cells were 86.88, 70.21, and 33.86 µm corresponding. In vivo and in vitro, AA in the cell microenvironment can be gradually oxidized into DHA.^[^ ^19^, ^70^, ^71^ ^]^ Hence, the H_2_O_2_ concentration released by DHA stimulation was higher than that of AA stimulation at the same concentration. This may be because DHA can directly act on PC12 cells, leading to the direct, rapid production of a large amount of H_2_O_2_, while AA may be gradually oxidized into DHA and then acts on cells to produce H_2_O_2_. Therefore, ethylenediaminetetraacetic acid disodium salt (EDTA‐2Na) was used to inhibit the oxidation of AA to DHA.^[^ ^72^, ^73^, ^74^ ^]^ The results showed that the H_2_O_2_ produced by cells decreased remarkably with EDTA‐2Na treatment (Figure 4E,F). Together, these results indicate that AA needs to be oxidized to DHA to produce H_2_O_2_. To prove this conclusion, the intracellular ROS levels of PC12 cells after different treatments, including blank medium, freshly configured AA solution, and DHA and AA solution containing EDTA‐2Na, were further analyzed. Figure 4I,J shows a very low intracellular ROS level in the cells treated with blank medium. However, the ROS levels of cells treated with fresh AA and DHA were higher than that of cells treated with blank medium. This indicates that AA and DHA caused cells to produce a large amount of ROS. ROS production caused by DHA was higher than that caused by AA, and when EDTA‐2Na was added to inhibit the oxidation of AA to DHA, the ROS level produced by AA stimulation decreased substantially. This suggests that DHA may cause ROS production. These results support the inference that AA enters the cells after oxidation to DHA, resulting in a dynamic imbalance of redox in the cells and the secretion of H_2_O_2_.

The specific molecular mechanism underlying the cellular production of H_2_O_2_ by DHA was further studied. DHA has a highly similar molecular structure to glucose (Figure S17, Supporting Information),^[^ ^75^ ^]^ thus, DHA may directly enter tumor cells via GLUT1 to stimulate H_2_O_2_ production.^[^ ^70^ ^]^Accordingly, high concentrations of glucose were first used to competitively bind to GLUT1, blocking DHA from entering cells through GLUT1. Figure 4G,H shows that H_2_O_2_ stimulated by DHA was substantially reduced after competitive binding to GLUT1. Then, GLUT1 was specifically blocked with BAY‐876, and the results showed that the production of H_2_O_2_ stimulated by DHA was further substantially reduced after specific blocking, the total H_2_O_2_ concentration produced by the tested cells is 86.88, 27.14, and 14.55 µm corresponding. Therefore, it can be concluded that DHA enters the cell via GLUT1 and stimulates the cell to produce H_2_O_2_.^[^ ^70^, ^75^ ^]^

We used a “Hydrogen Peroxide Assay Kit”, which can be used for the determination of H_2_O_2_ levels in cultured cells or tissues, as well as for the determination of H_2_O_2_ concentrations in the supernatant of cultured cells or in serum, urine, plasma, or other biological fluids to detect the production of H_2_O_2_ to further confirm the results of the sensor and conducted more in‐depth research on the process of DHA stimulating the secretion of H_2_O_2_ by cancer cells. Specifically, after stimulating HeLa cells with DHA, we did not use sensors to detect H_2_O_2_, but instead collected samples at the time point before stimulation (0 min) and at different time points after DHA stimulation, including 0.5, 1, 3, 5, and 10 min and then measured the absorbance at a wavelength of 560 nm using a spectrophotometer according to the instructions of the kit (Figure S18B, Supporting Information). Based on the obtained calibration curve, we calculated the H_2_O_2_ concentrations of the samples collected before and after stimulation (Figure S18A, Supporting Information). The average absorbance of the samples collected before stimulation is 0.029, corresponding to an H_2_O_2_ concentration of 3.5 µm, while the average absorbance of the samples collected after stimulation is 0.072, corresponding to a H_2_O_2_ concentration of 218.5 µm, which is similar to the results obtained by our sensor. Therefore, the analysis results of the H_2_O_2_ detection kit further confirm the results of the sensor and confirming once again the cell secretion of H_2_O_2_ stimulated by DHA is a fast process.

### 2.4 Mechanism Underlying the Ability of AA to Selectively Kill Cancer Cells and the Differential Therapeutic Effect of AAs

**Figure 5:**
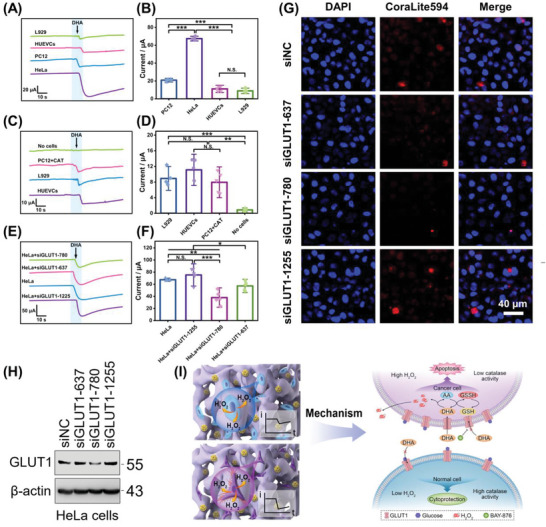
The mechanism of AA selective killing of cancer cells and the therapeutic differentiation of different cells. The current responses of PtNFs/CoPi@CC to the addition of 500 µm DHA to promote H_2_O_2_ release in A) the PBS containing non‐tumor cells (HUVECs, L929) and tumor cells (PC12, HeLa), respectively, and B) the statistical analysis of corresponding current responses, *n* = 5. The current responses of H_2_O_2_ from PBS containing non‐tumor cells (HUVECs and L929), PBS containing PC12 tumor cells and CAT, and PBS containing no cells (blank group) to the addition of 500 µm DHA to promote H_2_O_2_ release in C) the redox imbalance model and D) the statistical analysis of corresponding current responses, *n* = 5. The real‐time current response of H_2_O_2_ from HeLa cells to the addition of 500 µm DHA to promote H_2_O_2_ release in E) the GLUT1 knockdown model and F) the statistical analysis of corresponding current responses, *n* = 5. G) Confocal immunofluorescence images of the GLUT1 expression in HeLa cells under different conditions, including negative control (NC), siGLUT1‐637, siGLUT1‐780, and siGLUT1‐1255. Red: GLUT1, Blue: Nucleus. Scale bar: 40 µm. H) Immunoblot analysis of GLUT1 expression in HeLa cells under the condition of (G). I) Schematic illustration of the possible mechanism of AA selectively killing tumor cells. Data are shown as mean values ± 1.5 standard deviations. Two‐tailed *t*‐test was used for statistical analysis (N.S. means no significance, ^*^ *p* < 0.05, ^**^ *p* < 0.01, or ^***^ *p* < 0.001).

To explore the selective killing effect of AA on tumor cells and the reasons underlying its differential treatment effects, the differences in the release of H_2_O_2_ by DHA in various normal cells and tumor cells were first analyzed. **Figure** **5**A,B shows that the H_2_O_2_ concentration released by tumor cells under DHA stimulation was significantly higher than that released by normal cells, whereas the H_2_O_2_ released by HeLa cells was considerably higher than that released by PC12 cells, the total H_2_O_2_ concentration produced by the tested cells were 86.88, 262.29, 49.85, and 41.55 µm corresponding. Therefore, different tumor cells produce different amounts of H_2_O_2_ under DHA stimulation. Moreover, there was no significant difference in the H_2_O_2_ concentrations of the two types of normal cells stimulated by DHA. Many studies have demonstrated that tumor cells need to take in more glucose to maintain a high metabolic state due to their hallmarks of infinite proliferation and a shortened cell cycle.^[^ ^3^, ^4^ ^]^ Therefore, the expression of GLUT1 on the surface of tumor cells is higher than that of normal cells.^[^ ^76^ ^]^ Under stimulation with the same concentration of DHA, the DHA entering tumor cells through GLUT1 is substantially higher than that of normal cells, which leads to a greater concentration of H_2_O_2_ in tumor cells. Further experiments revealed that when a certain amount of CAT was used to treat PC12 cells, the H_2_O_2_ produced by DHA stimulation of PC12 cells was considerably reduced to the level of H_2_O_2_ produced by DHA stimulation of normal cells (Figure 5C,D), the total H_2_O_2_ concentration produced by the tested cells were 41.55, 49.85, 37.86, and 11.16 µm corresponding. This phenomenon may be a protective mechanism caused by a higher concentration of catalase in normal cells to prevent oxidative damage caused by intracellular redox disequilibrium.^[^ ^77^ ^]^

Considering that the research results were conducive to clinical application, HeLa cells were used in follow‐up experiments. Utilizing the gene interference technique, siRNA was transfected into HeLa cells to interfere with SLC2A1 gene expression (Figure S19 and Table S2, Supporting Information), thereby reducing GLUT1 expression on the cell membrane surface. Figure 5G shows that siGLUT1‐780 and siGLUT1‐637 reduced the expression of GLUT1 on the cell membrane. However, siGLUT1‐1255 had no significant reduction effect on GLUT1 expression. Western blot analysis also showed that siGLUT1‐780 and siGLUT1‐637 remarkably downregulated the expression of the SLC2A1 gene (Figure 5H), whereas siGLUT1‐1255 had a minimal interference effect. Importantly, DHA‐induced H_2_O_2_ release was substantially reduced after interference with GLUT1 expression on the membranes of HeLa cells (Figure 5E,F), the total H_2_O_2_ concentrations produced by the tested cells were 262.29, 292.01, 150.90, and 223.45 µm corresponding. Therefore, the amount of H_2_O_2_ released from tumor cells induced by DHA is proportional to the expression of GLUT1.

Taken together, these results provide novel insight into the molecular mechanism underlying the selective killing of cancer cells by AA and its differential killing effects on different types of cancer cells (Figure 5I). Specifically, DHA, the oxidative product of AA, enters cells through GLUT1 due to its structural similarity to glucose. It is then reconverted to AA through a non‐enzymatic reaction catalyzed by reduced glutathione (GSH) after entry into the cell, accompanied by the generation of oxidized glutathione (GSSH).^[^ ^71^, ^78^ ^]^ This causes dynamic disequilibrium of redox in cancer cells and leads to the production of excessive H_2_O_2_, resulting in apoptosis. However, normal cells are protected from the killing effect of AA due to the low GLUT1 number and the high intracellular catalase content. The above conclusions can well explain our experimental results that H_2_O_2_ levels will not increase with the passage of time after AA/DHA stimulation (Figure S18B, Supporting Information). However, according to the previous reports, after adding AA to the culture medium (without cells), H_2_O_2_ levels will time‐dependently increase.^[^ ^11^ ^]^ The important reason for the difference is that the production mechanism of H_2_O_2_ proposed in this manuscript is different from the previous reports. The previous reports have suggested that the most cogent explanation of AA in forming H_2_O_2_ as follows. The first step is for AA to lose an electron and form Asc^•−^. The electron reduces a protein‐centered metal: An example reaction is shown as reduction of Fe^3+^ to Fe^2+^. Fe^2+^ donates an electron to oxygen, forming active oxygen, including superoxide (O_2_^•−^) with subsequent dismutation to H_2_O_2_,^[^ ^10^, ^11^ ^]^ which is the production mechanism of extracellular H_2_O_2_ while what we proposed is the production mechanism of intracellular H_2_O_2_. Moreover, the differences in the production mechanism of H_2_O_2_ (intracellular/extracellular) mentioned above have been further validated in terms of reaction time. The time required for intracellular redox dynamics is relatively short (<30 s), while the time required for extracellular disproportionation reaction to produce H_2_O_2_ is relatively long (≈60 min to reach its maximum). In summary, our results expand the understanding of the use of AA in the treatment of cancer, and different production mechanisms of H_2_O_2_ may have more applications in the future due to potential synergistic effects.

## 3 Conclusion

This research preliminarily explored the molecular mechanism underlying the therapeutic effect of AA on tumors by constructing a new H_2_O_2_ detection platform. First, the MOF structure was grown on the conductive substrate by simple room‐temperature crystallization. Second, inspired by coral formation, a coral‐like CoPi array with a large specific surface area and multiple active sites was synthesized using the ion exchange method. This approach was safe and energy‐saving. Finally, PtNFs were uniformly electrodeposited on the surface of the array using the potentiostatic method. The constructed PtNFs/CoPi@CC sensor showed facile fabrication and great stability while maintaining excellent sensitivity and spatio‐temporal resolution. This enabled the real‐time observation of intracellular H_2_O_2_ release into the cellular microenvironment induced by AA and DHA. In addition, by combining this powerful H_2_O_2_ sensor with inhibitor‐base study, catalase, and gene interference techniques, our results demonstrated that the release of H_2_O_2_ after GLUT1‐mediated DHA stimulation induced dynamic redox disequilibrium in cancer cells. Moreover, the results confirmed that this release was positively correlated with the number and activity of GLUT1 on the cell membrane. These findings provide insight into the molecular mechanism underlying AA's selective killing of cancer cells and its differential killing effect on different cancer cells. The approach adopted here for synthesizing unique structures inspired by nature and biomimetic ideas will expand the horizon of materials development in the fields of sensing and catalysis. Moreover, this work provides a robust tool for determining the molecular information of oxidative stress in tumor cells. Thus, it holds great promise for providing insight into the anticancer molecular mechanism of AA and the development of potential therapeutic strategies. The novel sensing platform described here has potential application value in cancer pathology research in the future, and can serve as a supplementary procedure for the discovery of anticancer drugs. However, due to the size limitations of the 2D sensing platform, its clinical application in tumor tissues and living organisms is potentially challenging. Further work in this space is a critical direction of future research.

## 4 Experimental Section

### Preparation of PtNFs/CoPi@CC

As shown in Figure S20 (Supporting Information), first, commercial flexible carbon cloth (CC, 1 × 1 cm^2^) was treated with ultrasonic waves in acetone, ethanol, and ultra‐pure water for 2 min to remove impurities. After cleaning, the CC was soaked in a mixture of 1:1 concentrated nitric acid and sulfuric acid, and heated and boiled for 1 h to improve the hydrophilicity of the CC. After cooling, it was rinsed with plenty of ultra‐pure water until deacidified and the pH of the eluent, as measured by a pH meter was 7.00. After cleaning, the CC was dried in a vacuum drying oven at 60 °C for reserve. Scissors were used to cut the commercial CC into a hammer CC electrode with an effective area of 1 × 1 cm. Then, 2‐Methylimidazole (2‐MIM) aqueous solution (10 mL, 0.4 m) was quickly poured into cobalt nitrate hexahydrate (Co(NO_3_)_2_·6H_2_O) aqueous solution (10 mL, 0.05 m) under a state of agitation. After fully stirring for 5 min, the CC trimmed after pretreatment was soaked in the mixture and stood for 4 h. Co‐MOF was grown on the CC by the room temperature crystallization method. After taking out the CC, it was washed with ultra‐pure water and then dried in a vacuum drying oven at 60 °C for 1 h to obtain Co‐MOF@CC. Then, the prepared Co‐MOF@CC was etched in PBS (pH 7.40, 20 mL, 0.1 m) at room temperature for 24 h to achieve the conversion of Co‐MOF to CoPi on the CC conductive substrate. Next, the prepared electrode was cleaned several times with ultra‐pure water and dried in a 60 °C oven to obtain the CoPi@CC electrode. The CoPi@CC obtained by fixing on a platinum‐clip electrode was deposited into the electrodeposition liquid containing 0.01 m Na_2_SO_4_ and 2 mm H_2_PtCl_6_ and adjusted to neutral pH (7.00) by NaOH. The electrodeposition was performed for 4800 s at a working voltage of −0.2 V by *i–t*. The PtNFs/CoPi@CC sensor electrode was successfully prepared after cleaning with ultra‐pure water.

### In Situ Detection of Cell Secretion of H_2_O_2_

L929, HUEVCs, PC12, and HeLa cells were all purchased from iCell Bioscience Inc. (Shanghai, China). L929, PC12, and HUEVCs were cultured in 1640 medium and HeLa cells were cultured in DMEM medium containing 10% fetal bovine serum (FBS) and 1% penicillin‐streptomycin at 5% CO_2_ and 37 °C. After the cells were inoculated in cell culture dishes for 24 h, the original medium was removed and 3 mL complete culture medium containing FBS mentioned above containing 5 g L^−1^ glucose, 100 µm BAY‐876, or 500 U mL^−1^ CAT was added, respectively; and 3 mL of cell‐free complete culture medium containing FBS was used for the control group. Cells were incubated with BAY‐876 and glucose for 10 min. Then, the sterile PtNFs/CoPi@CC obtained after ultraviolet irradiation was used to measure the current response with *i–t* at the optimal applied potential (−0.5 V). In the detection process, after the signal stabilized, 500 µm AA, AA+EDTA‐2Na, or DHA were used as stimulants to detect the release of H_2_O_2_ from cells.

### Statistical Analysis

Statistical analysis was performed using Excel and GraphPad Prism 9. The sample size for each statistical analysis was 3 or 5 (*n* = 3 or 5). All data in this study were expressed as the mean ± 1.5 standard deviation (SD). Two‐tailed *t*‐tests were used for the statistical comparison of groups. Significance was expressed as ^*^ *p* < 0.05, ^**^ *p* < 0.01, or ^***^ *p* < 0.001. N.S. means no statistically significant difference.

## Conflict of Interest

The authors declare no conflict of interest.

## Supporting information

Supporting Information

Supplemental Video 1

Supplemental Video 2

## Data Availability

Research data are not shared.
