# Supplementary material for: A Bioinspired Flexible Sensor for Electrochemical Probing of Dynamic Redox Disequilibrium in Cancer Cells
Source: Adv Sci (Weinh). 2023 Nov 9;10(36):2304079. doi: 10.1002/advs.202304079 (PMC10754098; doi:10.1002/advs.202304079)
Supplement: Supplementary file 1 — Supporting Information [file ADVS-10-2304079-s001.pdf]

ADVANCED SCIENCE
Open Access

Supporting Information

for *Adv. Sci.*, DOI 10.1002/advs.202304079

A Bioinspired Flexible Sensor for Electrochemical Probing of Dynamic Redox Disequilibrium in Cancer Cells

Zhongyuan Zeng, Jian Wang, Shuang Zhao, Yuchan Zhang, Jingchuan Fan, Hui Wu, Jiajia Chen, Zaikuan Zhang, Zexuan Meng, Lu Yang, Renzhi Wang, Bo Zhang, Guixue Wang, Chen-Zhong Li* and Guangchao Zang**

Supporting Information

A Bioinspired Flexible Sensor for Electrochemically Probing of Dynamic Redox Disequilibrium in Cancer Cells

Zhongyuan Zeng, Jian Wang, Shuang Zhao, Yuchan Zhang, Jingchuan Fan, Hui Wu, Jiajia Chen, Zaikuan Zhang, Zexuan Meng, Lu Yang, Renzhi Wang, Bo Zhang, Guixue Wang, Chen-zhong Li*, Guangchao Zang**

1. Experiments and Methods

1.1. Chemicals and Reagents

1.2. Instruments

1.3. Electrochemical Measurements

1.4. Living/Dead Cell Fluorescent Staining

1.5. Cytotoxicity Test (CCK-8 Assay)

1.6. Cell transfections

1.7. Intracellular ROS levels

1.8. Western blotting analysis

1.9. Immunofluorescence assay

Supplemental Figures

Figure S1. The SEM images of the construction process of detection platform

Figure S2. The SEM Images of electrodes with incomplete ion exchange reaction

Figure S3. The analysis of HRTEM image of PtNFs/CoPi@CC

Figure S4. The detailed process of measuring lattice spacing

Figure S5. EDS mapping of PtNFs/CoPi@CC

Figure S6. The simulated volume models

Figure S7. The crystal structures

Figure S8. Optimization of electrodeposition time and applied potential

Figure S9. The current response recorded by CVs and the corresponding calibration curve

Figure S10. Changes in conductivity during electrode modification process

Figure S11. Study of the dynamic process on the surface of PtNFs/CoPi@CC

Figure S12. Selectivity of PtNFs/CoPi@CC toward H2O2

Figure S13. Study on the stability of the electrodes

Figure S14. The study of the reproducibility and stability of PtNFs/CoPi@CC

Figure S15. The biocompatibility of PtNFs/CoPi@CC

Figure S16. The Performance of PtNFs/CoPi@CC in complex environments.

Figure S17. The chemical structural formula

Figure S18. Verification of results of the sensor by hydrogen peroxide assay kit.

Figure S19. Record the process of fluorescence (FAM) labeled siRNA transfection into HeLa cells

Figure S20. Photographs of the construction process of detection platform

Supplemental Tables

Table S1. Comparison of a proposed catalyst with reported materials for the H_2O_2 sensors

Table S2. Summary of the siRNA sequences used in the study

Supplemental Notes

Note S1. The synthesis mechanism of Co-MOF and cobalt phosphate (CoPi)

Note S2. The corresponding chemical structural formula

Supplemental References

1. Experiments and methods

1.1. Chemicals and Reagents

The commercial CC (Ce Tech Co., Ltd) was purchased from Hubei Rocktek Instrument Co., Ltd. (Wuhan, China). Acetone, ethanol, sulfuric acid (H_2SO_4), nitric acid (HNO_3), disodium hydrogen phosphate dodecahydrate ($Na_2HPO_4·12H_2O$), sodium dihydrogen phosphate dihydrate ($NaH_2PO_4·2H_2O$) were obtained from Chongqing Chuandong Chemical Group Co., Ltd (Chongqing, China). 2-Methylimidazole (2-MIM), catalase (from bovine liver), Dopamine (DA), uric acid (UA), ascorbate acid (AA), adrenaline (E), norepinephrine (NE), 3,4-dihydroxyphenylacetic acid (DOPAC), 5-hydroxytryptamine (5-HT) were purchased from Adamas-beta (Shanghai Titan Scientific Co., Ltd, China). Phosphate buffer solution (PBS) was prepared by mixing $Na_2HPO_4·12H_2O$ (0.1 M) and $NaH_2PO_4·2H_2O$) (0.1 M) at a ratio of 81:19, and the pH was adjusted to 7.40 using $Na_2HPO_4·12H_2O$ (0.1 M) or $NaH_2PO_4·2H_2O$ (0.1 M) by pH meter. Hydrogen peroxide solution (30 wt%, 9.79M) was purchased from Kelong Chemical Reagent Factory (Chengdu, China). Potassium ferrocyanide trihydrate ($K_4[Fe(CN)_6]$) and potassium ferricyanide ($K_3[Fe(CN)_6]$) were purchased from Aladdin Reagent Co., Ltd (Shanghai, China). Potassium ferricyanide solution ($[Fe(CN)_6]^{3-/4-}$)was prepared with the PBS containing 5 mM $K_3[Fe(CN)_6]$, 5 mM $K_4[Fe(CN)_6]$) and 0.1 M KCl. BAY-876 was purchased from TargetMol (Shanghai, China). Glucose and potassium chloride (KCl) were purchased from Jiangsu Qiangsheng Functional Chemical Co., Ltd (Jiangsu, China). Cobalt (II) nitrate hexahydrate ($Co(NO_3)_2·6H_2O$), ethylenediaminetetraacetic acid disodium salt (EDTA-2Na), sodium hydroxide (NaOH), chloroplatinic acid hexahydrate ($H_2PtCl_6·6H_2O$) and sodium sulfate anhydrous (Na_2SO_4) were purchased from Sangon Biotech Co., Ltd (Shanghai, China), Ascorbate oxidase (AAox) was purchased from Sigma-Aldrich (St. Louis, MO, USA). Ultrapure water (18.2 MΩ) was produced by a ultrapure water system (AOSIDE INSTRUMENT) and used throughout the experiment. RPMI Medium 1640 basic and Dulbecco's Modified Eagle Medium were purchased from Gibco. Fetal bovine serum (FBS) was provided by Lonsera. CCK-8 and Calcein/PI Cell Viability/Cytotoxicity Assay Kit, and Hydrogen Peroxide Assay Kit (S0038) were purchased from Beyotime Biotechnology.

1.2. Instruments

The morphology of PtNFs/CoPi/@CC was observed by field emission scanning electron microscope (S-8010, Hitachi, Japan). Energy Dispersive X-ray spectroscopy (EDX) images of PtNFs/CoPi@CC were obtained by using X-MaxN (Oxford Instruments, UK). X-ray photoelectron spectroscopy (XPS) measurements were performed with a photoelectron spectrometer (K-Alpha,

Thermo Fisher Science). The X-ray diffraction pattern of PtNFs/CoPi/@CC was obtained by X-ray diffractometer (XRD-6100, SHIMADZU). Fluorescence imaging was performed by using an inverted fluorescence microscope (ECLIPSE Ti2, Nikon). Transmission electron microscopy (TEM) images were recorded in JEM-2100F operated at 200 kV (Jeol Instrument, Japan). Fourier Transform infrared spectroscopy (FT-IR) was obtained by the Thermo Scientific™Nocolet™ iS™5 FT-IR spectrometer and Thermo Scientific™iD7 ATR annex. The absorbance was measured by a spectrophotometer (Thermo Scientific™ Multiskan GO 1510, Finland).

1.3. Electrochemical Measurements

All electrochemical measurements were performed using the electrochemical workstation (CHI 660E, Shanghai, China). A classical three-electrode system was constructed with Pt wire as the counter electrode, Ag/AgCl electrode as the reference electrode and PtNFs/CoPi@CC as the working electrode. In a 5 mM potassium ferriccyanide solution containing 0.1 M KCl, the modified electrode was characterized by CV and EIS. The conditions for CV characterization were: potential range -0.1 V to 0.6 V, sweep rate 100 mV/s, EIS spectrum frequency range 100 kHz to 0.1 Hz. The i-t, CV and DPV were carried out in 0.1 M PBS solution (pH=7.40). The optimal working voltage was -0.5 V. The working voltage range of CV was -1.0 V to 1.0 V, and the scanning speed was 100 mV/s. DPV has a voltage range of -1.0 V to 1.0 V, a pulse amplitude of 50 mV, and a pulse period of 0.5 s.

1.4. Living/Dead Cell Fluorescent Staining

PC12 cells were purchased from icell Bioscience Inc. (Shanghai, China). PC12 cells were cultured in 1640 medium containing 10% fetal bovine serum and 1% penicillin-streptomycin. PC12 cells grew on PtNFs/CoPi@CC electrode were fluorescentially stained with Calcein-AM/PI double staining kit to observe the growth of PC12 cells on PtNFs/CoPi@CC electrode and evaluated the biocompatibility of the electrode. Specifically, diluted Calcein AM and PI with the buffer solution at a ratio of 1:1000 to configure the Calcein AM/PI detection working liquid. The cells were planted on PtNFS/CoPi@CC in 24-well plate for 18 hours, washed with PBS. Then 250 μL Calcein-AM/PI detection solution was added to each well and incubated at 37 °C in the dark for 30 minutes to stain. Finally, the growth of PC12 cells on PtNFs/CoPi@CC was recorded under a fluorescence microscope.

1.5. Cytotoxicity Test (CCK-8 Assay)

The PtNFs/CoPi@CC was washed three times in ultra-pure water and cut into a suitable size, then exposed to ultraviolet light (UV) for disinfection. PC12 cells were digested and then suspended, inoculated in a 96-well plate for 8 hours, and then CC pieces were added to the holes. After incubating

for 8 h, the culture medium was removed, and fresh culture medium (100 μL) containing CCK-8 (10 μL) was added to each well. The absorbance at 450 nm was recorded when the color of the solution turned orange for 2 h. Relative cell survival was calculated according to the following formula: $(OD_{test}/OD_{Control})\times100\%$.

1.6. Cell transfections

HeLa cells at 60-80% confluence were transfected with four kinds of siRNA, including siNC, siGLUT1-637, siGLUT1-780 and siGLUT1-1255 (GenePharma) using siRNA Mate Transfection Reagent (GenePharma) according to the manufacturer's protocol.

1.7. Intracellular ROS levels

In order to verify the amount of reactive oxygen species produced by cells induced by different treatment conditions, PC12 cells were inoculated in petri dishes, cultured in 37 °C, 5% CO_2 and humid environment for 24 hours, incubated for 30 min with DCFH-DA (Beyotime Biotechnology) at 37 °C, and then freshly configured ascorbic acid solution with a final concentration of 500 μM, fresh AA solution containing EDTA-2Na and dehydroascorbic acid (DHA) solution were added for 5 minutes. Inverted fluorescence microscope was used for imaging observation and analysis (ECLIPSE Ti2, Nikon).

1.8. Western blotting analysis

First, an appropriate amount of RIPA cell lysate was added to HeLa cells, then reversed lysate for 20-30 minutes and centrifuge to collect the supernatant. Protein concentrations were then determined using a BCA kit (Beyotime Biotechnology). Finally, the protein was isolated by SDS-PAGE gel and transferred to PVDF membrane. Membranes were blocked in nonfat powdered milk (5%) for 1 h. Target protein was detected using specific primary antibody (GLUT1, Santa cruz biotechnology, sc-377228; β-actin, Proteintech) and incubated at 4 °C overnight. Bound antibodies were detected by horseradish-peroxidase-conjugated secondary antibody and visualized by ECL Plus Kit (Meilunbio).

1.9. Immunofluorescence assay

The treated cells were washed with PBS for 5 minutes and repeated three times. After washing, fixed with 4% paraformaldehyde at room temperature for 20 min, and then treated with closed buffer solution (3% BSA) at room temperature for 30 min. After closure, GLUT1 antibody was added and incubated at 4 °C overnight. After that, CoraLite594 labeled secondary antibody (Proteintech) was added and incubated at room temperature for 50 min. After cleaning, the tablets were sealed with anti-fluorescence attenuation sealing tablet containing DAPI (Solarbio). Finally, NCSU-1 laser scanning

confocal microscope (LSCM, Yongxin, China) and NIB900 inverted fluorescence microscope (Nexcope, Yongxin, China) were used for imaging observation.

Supplemental Figures

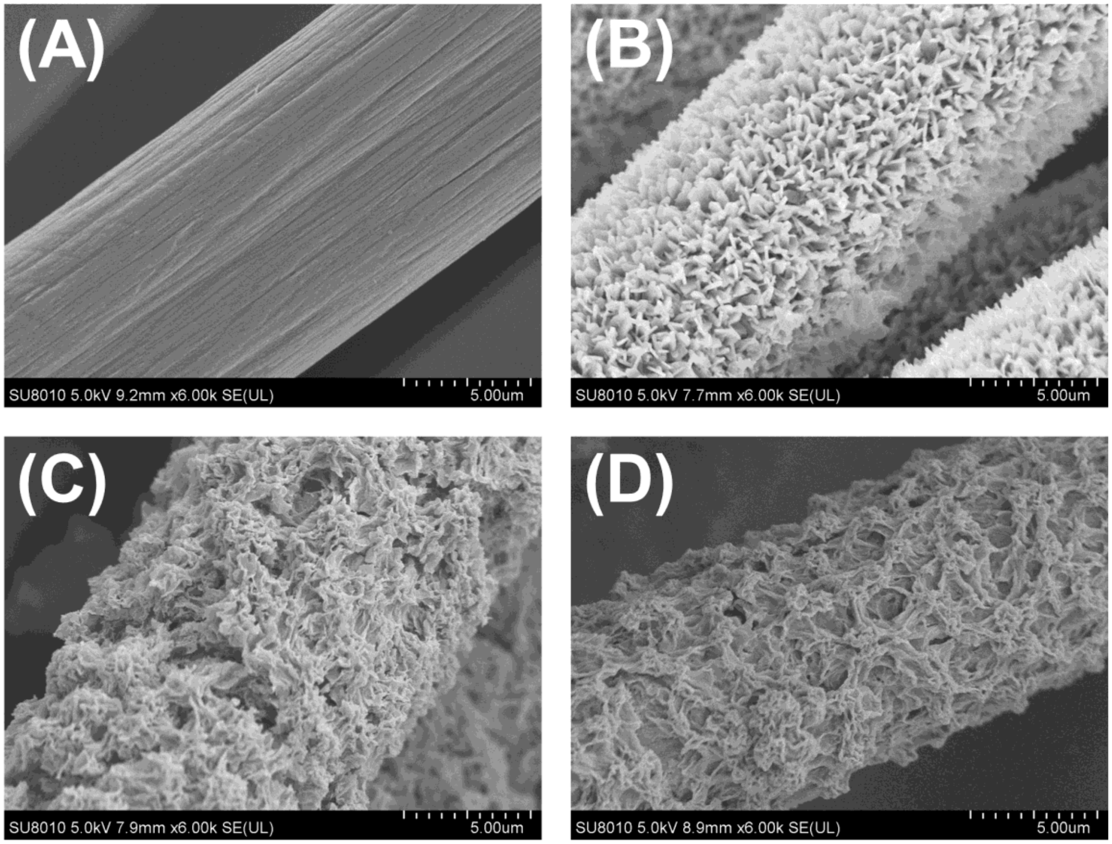

Figure S1. The SEM images of the construction process of detection platform. SEM images of electrodes: (A) bare CC, (B) Co-MOF@CC, (C) CoPi@CC and (D) PtNFs/CoPi@CC. Scale bar=5 μm.

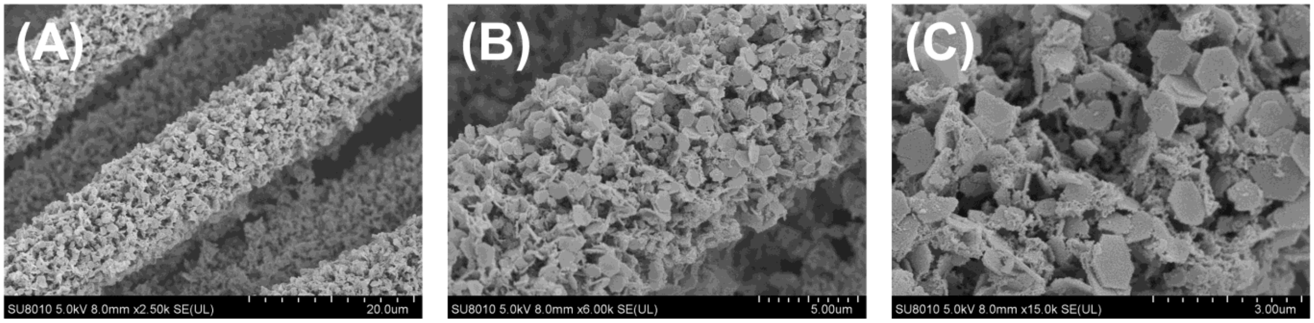

Figure S2. The SEM Images of electrodes with incomplete ion exchange reaction. SEM images of Co-MOF@CC electrodes after etching for 12 hours, which modified by the intermediates consist of Co-MOF and CoPi namely Co-MOF/CoPi@CC. (A) 20 μm, (B) 5 μm and (C) 3 μm.

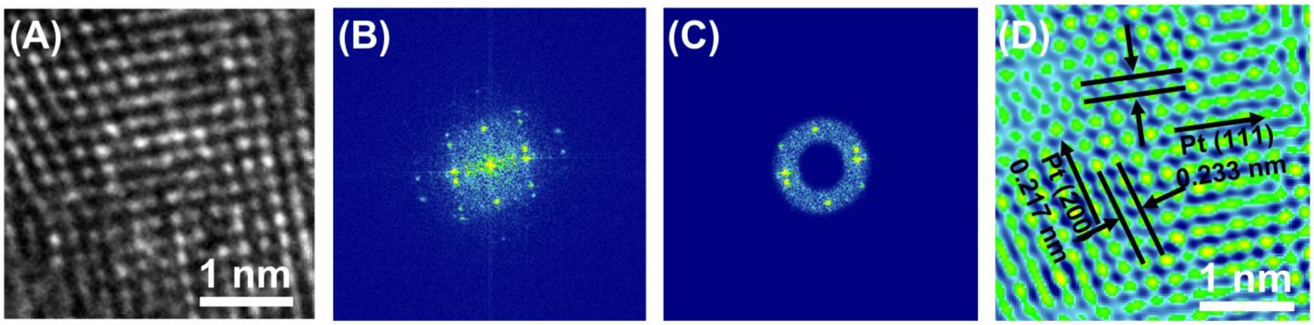

Figure S3. The analysis of HRTEM image of PtNFs/CoPi@CC. (A) HRTEM image of PtNFs/CoPi@CC and (B) corresponding fast Fourier transformation (FFT) images. (C) corresponding FFT images of (A) after apply mask pattern. (D) corresponding inverse fast Fourier transformation (IFFT) images and the analysis of lattice.

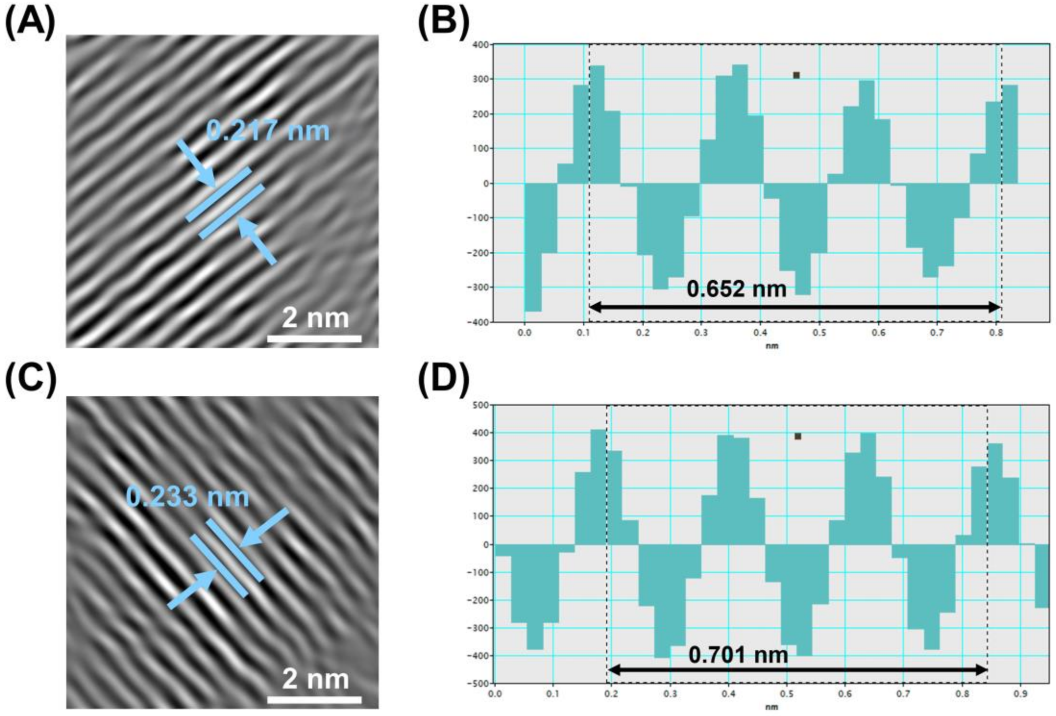

Figure S4. The detailed process of measuring lattice spacing. The inverse fast Fourier transformation (IFFT) images of PtNFs/CoPi@CC (A) Pt (200) crystal planes and (B) the detail of measurement of corresponding lattice distance of (A). (C) Pt (111) crystal planes and (D) the detail of measurement of corresponding lattice distance of (C).

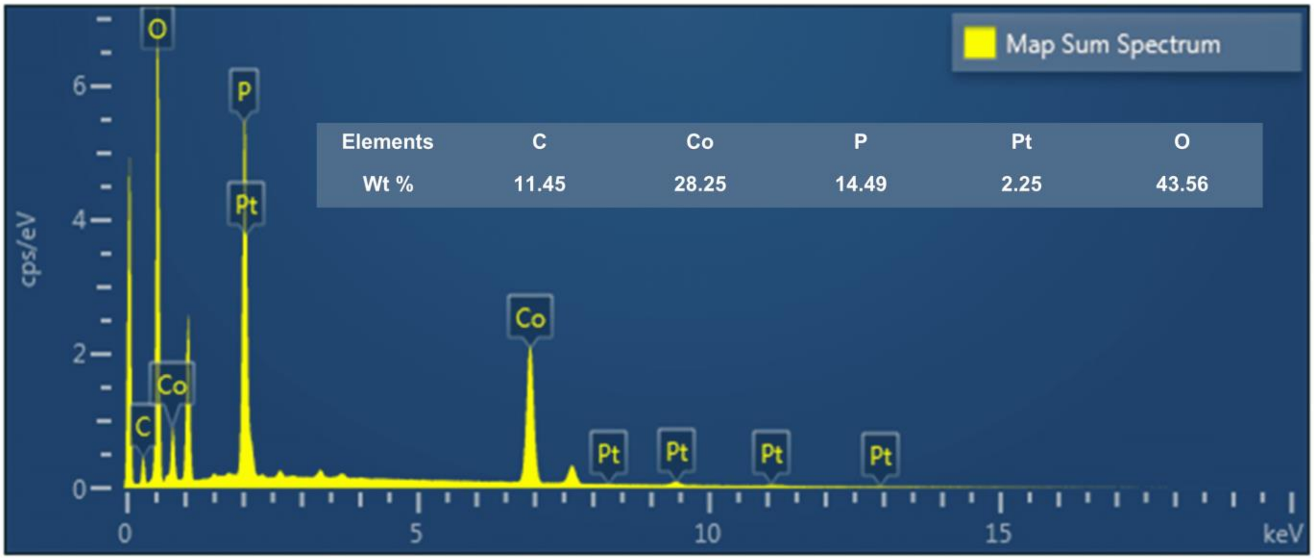

Figure S5. EDS mapping of PtNFs/CoPi@CC.

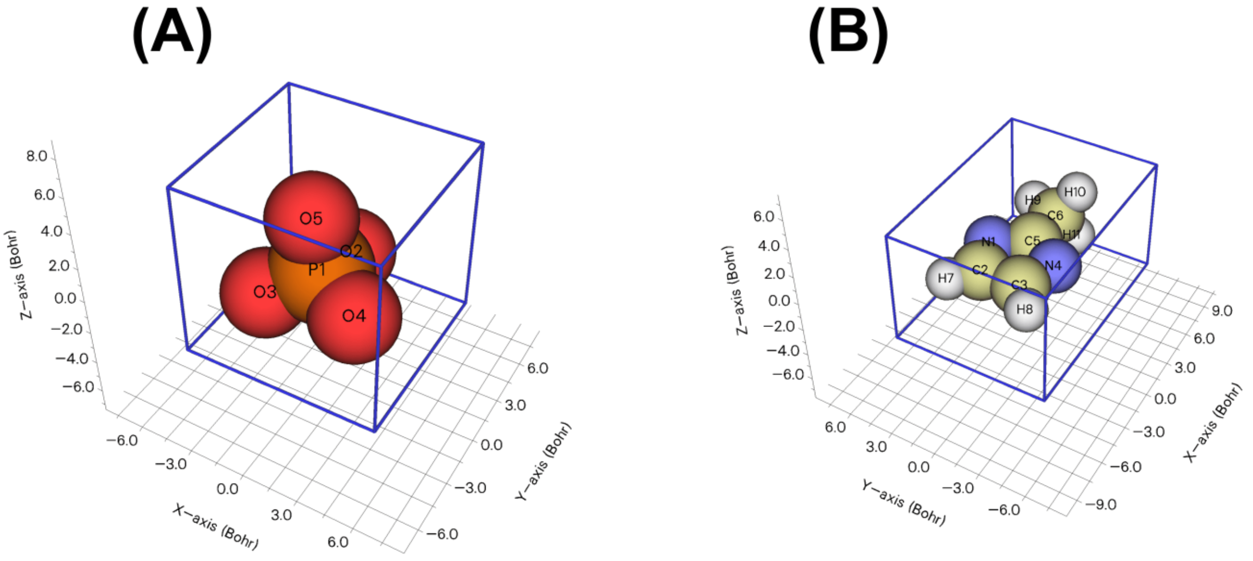

Figure S6. The simulated volume models. (A) Phosphate radical and (B) deprotonated 2-MIM.

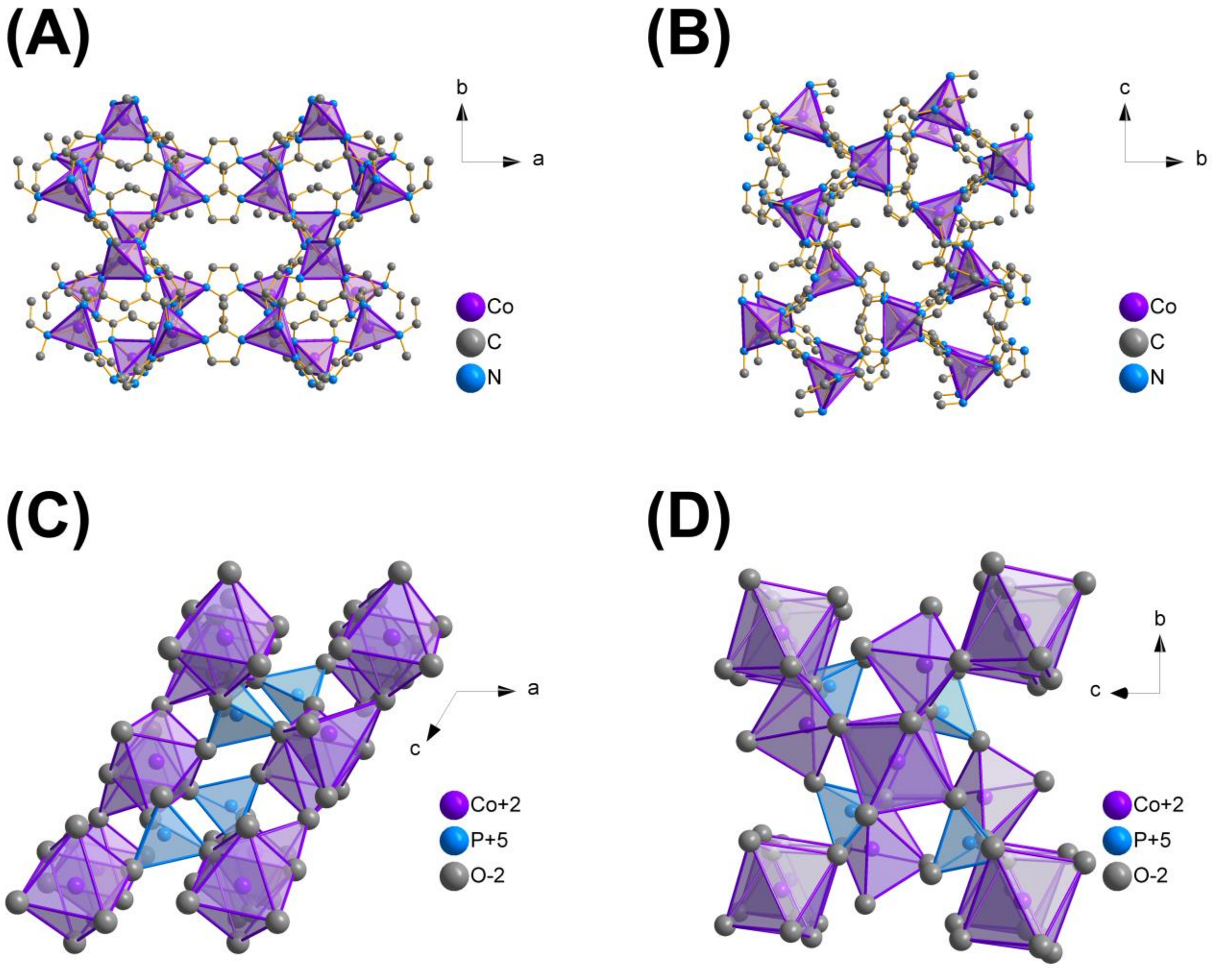

Figure S7. The crystal structures. The crystal structure of Co-MOF from different viewing angle (A) and (B), and the crystal structure of CoPi from different viewing angle (C) and (D).

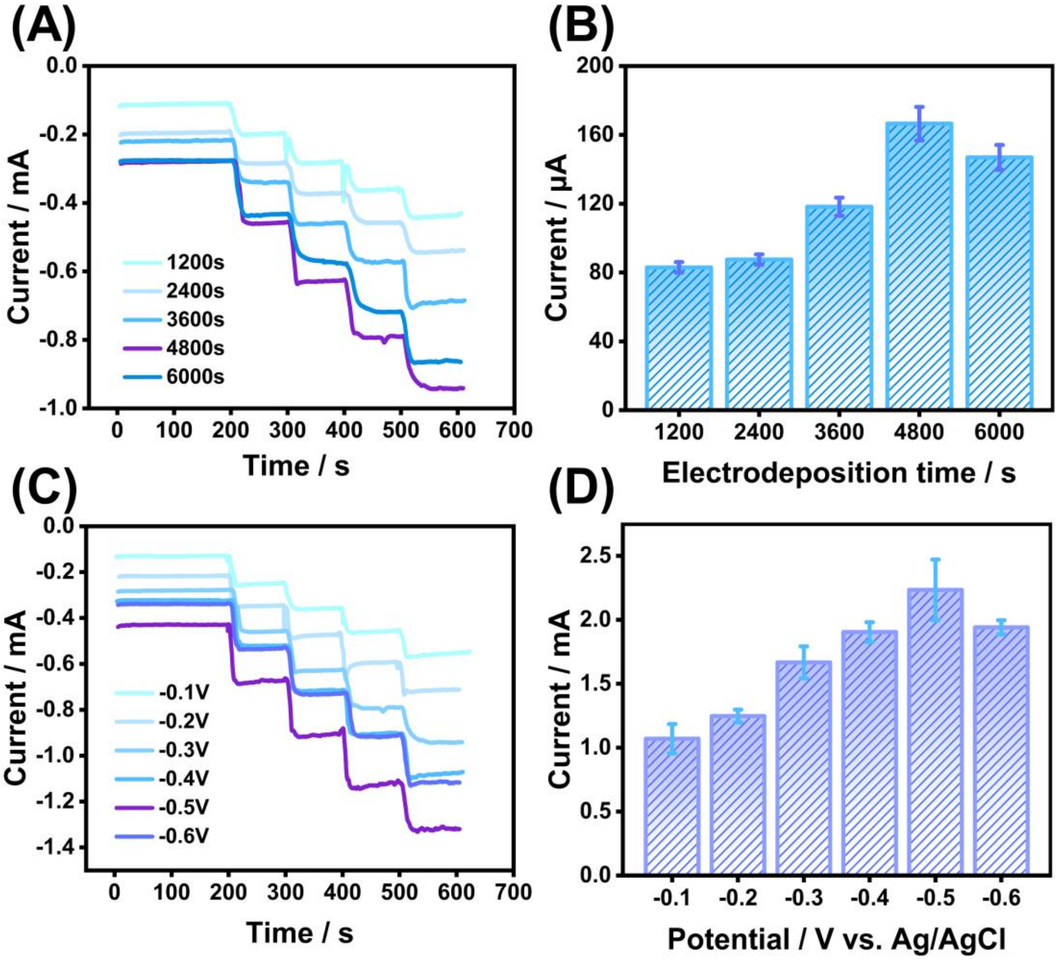

Figure S8. Optimization of electrodeposition time and applied potential. Amperometric current curves of the PtNFs/CoPi@CC under different electrodeposition times with the successive addition of H_2O_2 into the stirring 0.1 M PBS, pH=7.40 (A) and corresponding statistical results (B). Amperometric current curves of the PtNFs/CoPi@CC with the successive addition of H_2O_2 into the stirring 0.1 M PBS (pH=7.40) at different potentials (C) and corresponding statistical results (D). Error bars are the standard error of the mean (n=4).

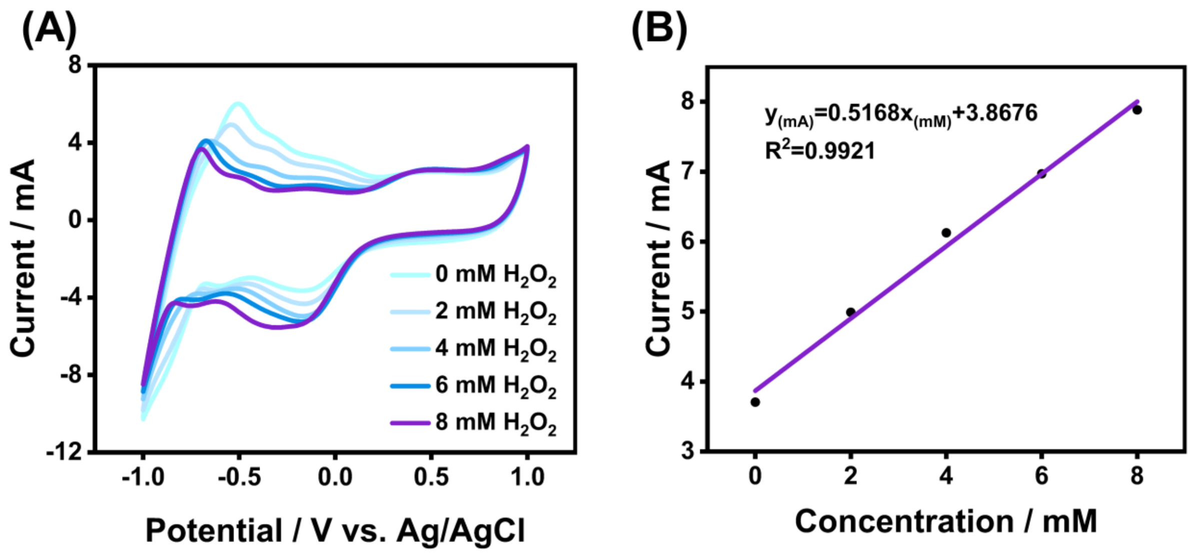

Figure S9. The current response recorded by CVs and the corresponding calibration curve. (A) CVs of PtNFs/CoPi@CC electrode in 0.1 M PBS solution at a scan rate of 100 mV·s^{-1} in the absence (0 mM H_2O_2) and presence of different concentrations (2, 4, 6, 8 mM) of H_2O_2. (B) The calibration curve of peak cathodic current with different H_2O_2 concentrations.

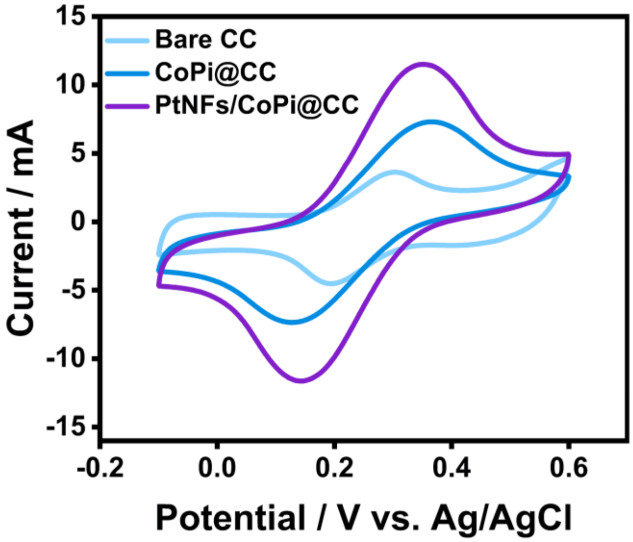

Figure S10. Changes in conductivity during electrode modification process. CVs of bare CC, CoPi@CC and PtNFs/CoPi@CC electrodes in 5 mM $[Fe(CN)_6]^{3-/4-}$ containing 0.1 M KCl solution at scan rate of 100 $mV \cdot s^{-1}$.

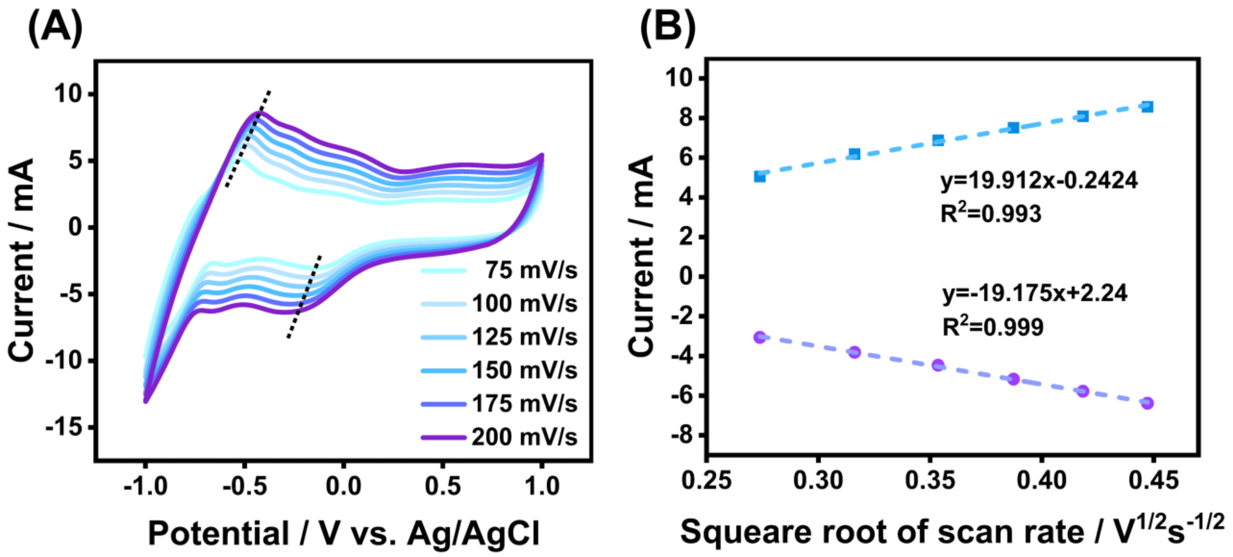

Figure S11. Study of the dynamic process on the surface of PtNFs/CoPi@CC. (A) CVs of the PtNFs/CoPi@CC at different scan rates. (B) The corresponding plot of current vs. square root of scan rate. All experiments were performed in 0.1 M PBS (pH=7.40).

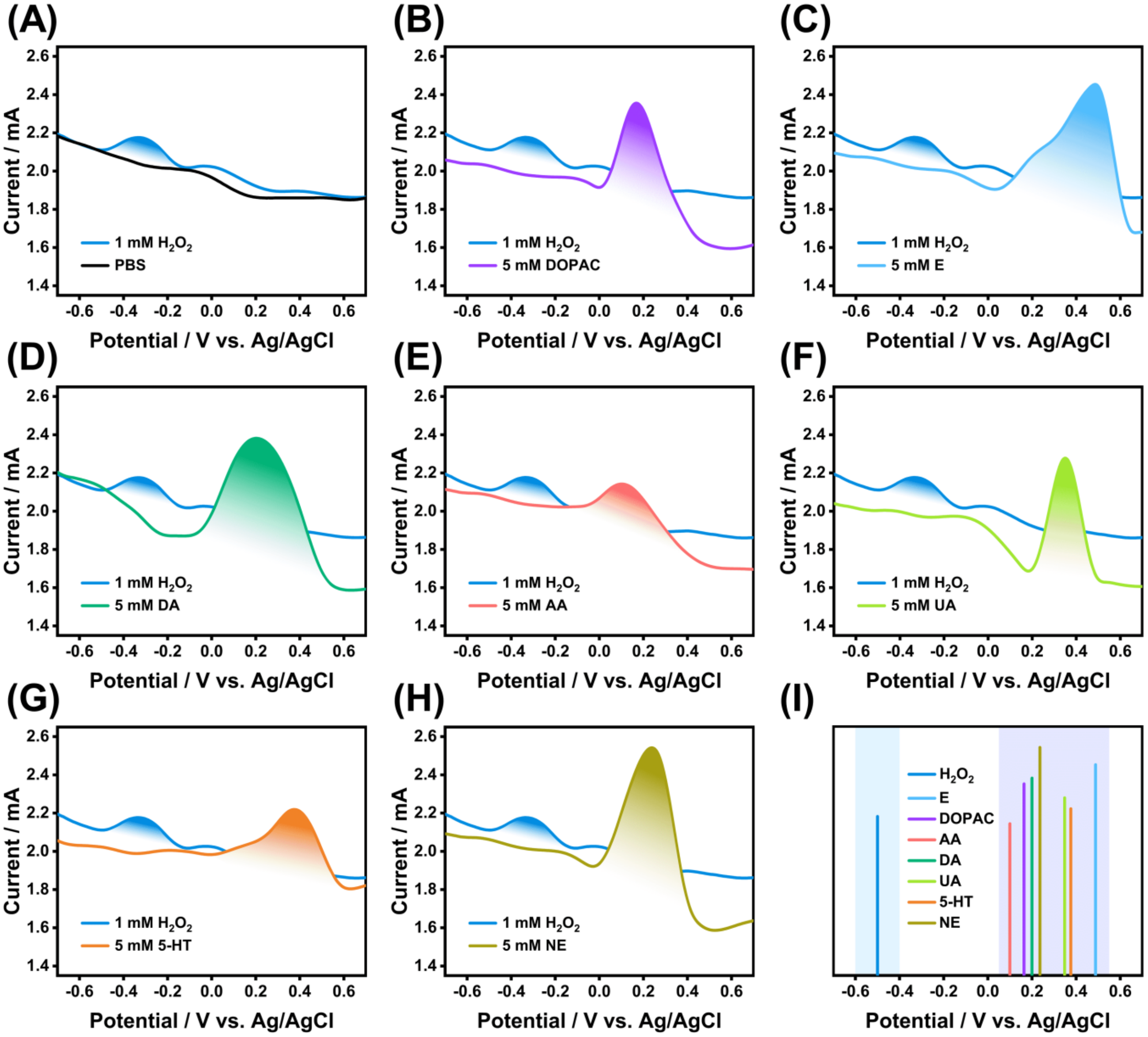

Figure S12. Selectivity of PtNFs/CoPi@CC toward H_2O_2. DPVs images of 1.0 mM H_2O_2 with (A) PBS, (B) 5 mM DOPAC, (C) 5 mM E, (D) 5 mM DA, (E) 5 mM AA, (F) 5 mM UA, (G) 5 mM 5-HT and (H) 5 mM NE at PtNFs/CoPi@CC. (I) The different peak potentials (-0.5 V, 0.1 V, 0.164 V, 0.2 V, 0.236 V, 0.348 V, 0.376 V, 0.488 V) were marked by lines using corresponding color, respectively.

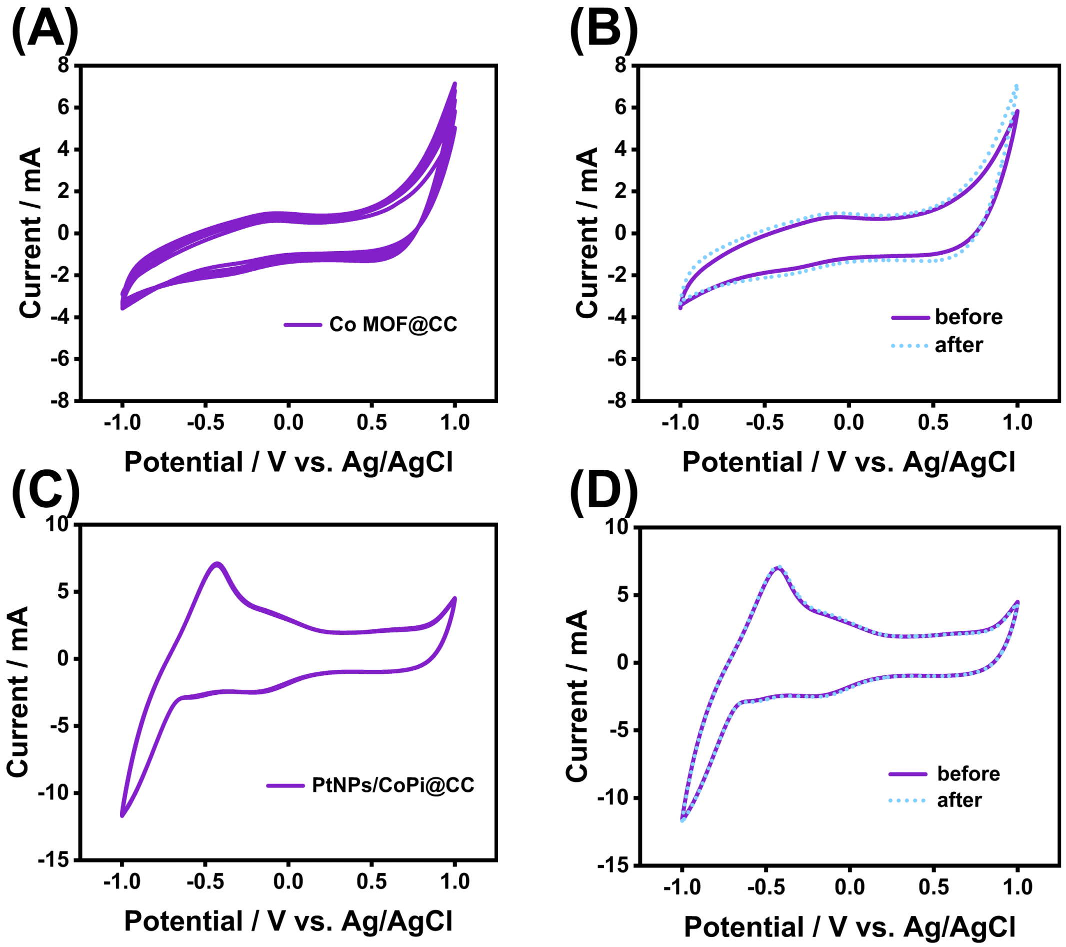

Figure S13. Study on the stability of the electrodes. (A) CVs of Co-MOF@CC during 5 cycles and (B) the 1st CV cycle comparison with the 5th CV cycle of Co-MOF@CC. (C) CVs of PtNFs/CoPi@CC during 5 cycles and (D) the 1st CV cycle comparison with the 5th CV cycle of PtNFs/CoPi@CC.

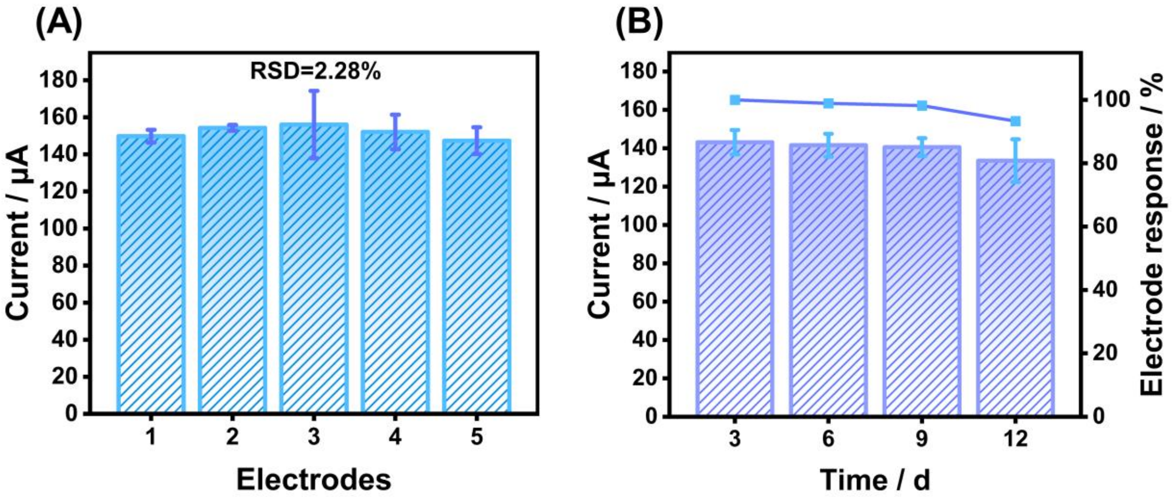

Figure S14. The study of the reproducibility and stability of PtNFs/CoPi@CC. Amperometric responses of the PtNFs/CoPi@CC electrode to the successive dropwise additions of 0.5 mM H_2O_2 in 0.1 M PBS (pH=7.40), n=4; the reproducibility (A) and stability (B) of PtNFs/CoPi@CC electrode for H_2O_2 detection, and the amperometric responses to H_2O_2 were 100% (3 days), 98.9% (6 days), 98.2% (9 days) and 93.3% (12 days), respectively. Error bars are the standard error of the mean (n=4).

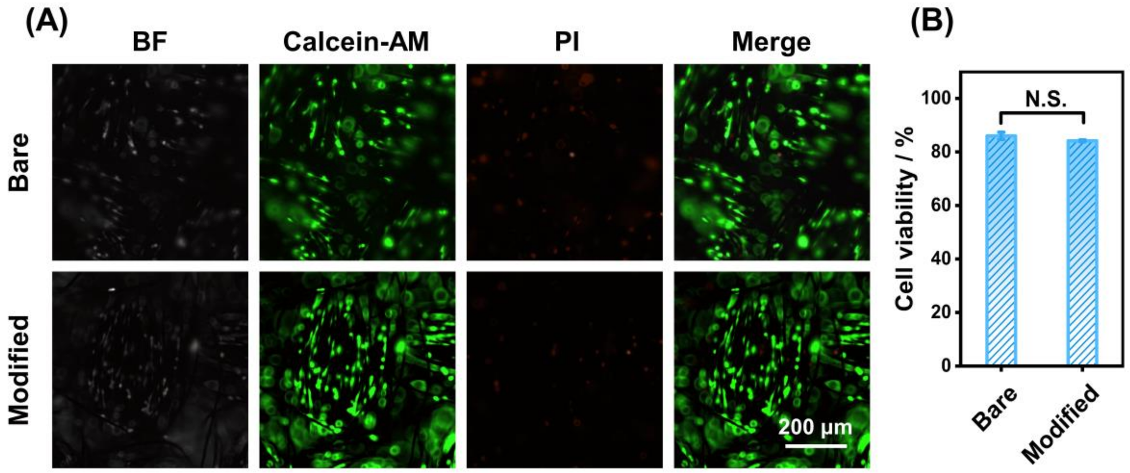

Figure S15. The biocompatibility of PtNFs/CoPi@CC. (A) Bright-field and fluorescence imaging of PC12 cells stained with Calcein-AM (green) and PI (red). (B) Cell viability of PC12 incubated with PtNFs/CoPi@CC (Modified) and CC (Bare) for 8 h. Error bars are the standard error of the mean (n=3).

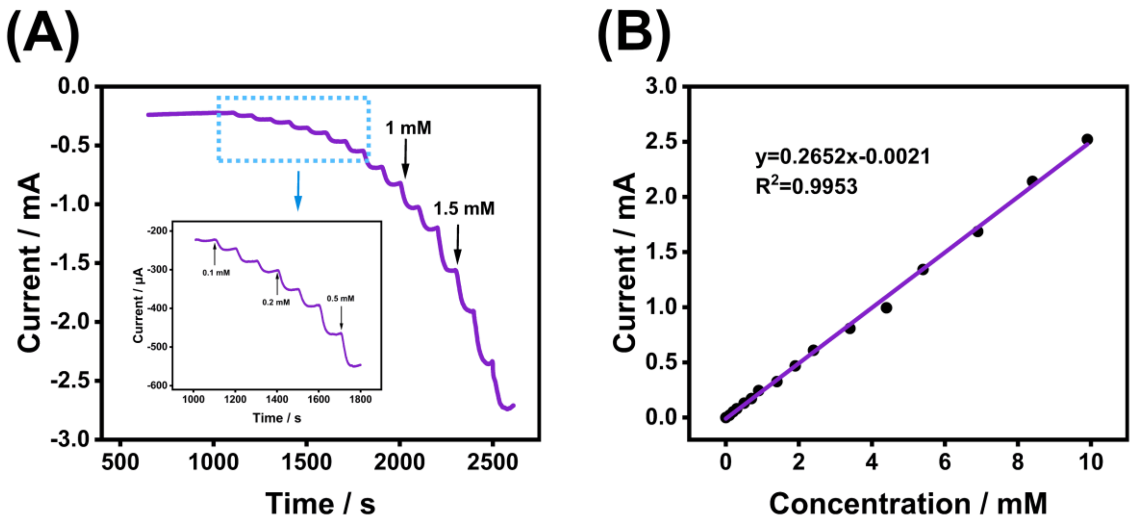

Figure S16. Amperometric response of PtNFs/CoPi@CC in a complete culture medium containing fetal bovine serum at -0.5 V upon successive additions of H_2O_2 from 100 μM to 1.5 mM (triple injections per concentration) (A) and (B) the corresponding calibration curves.

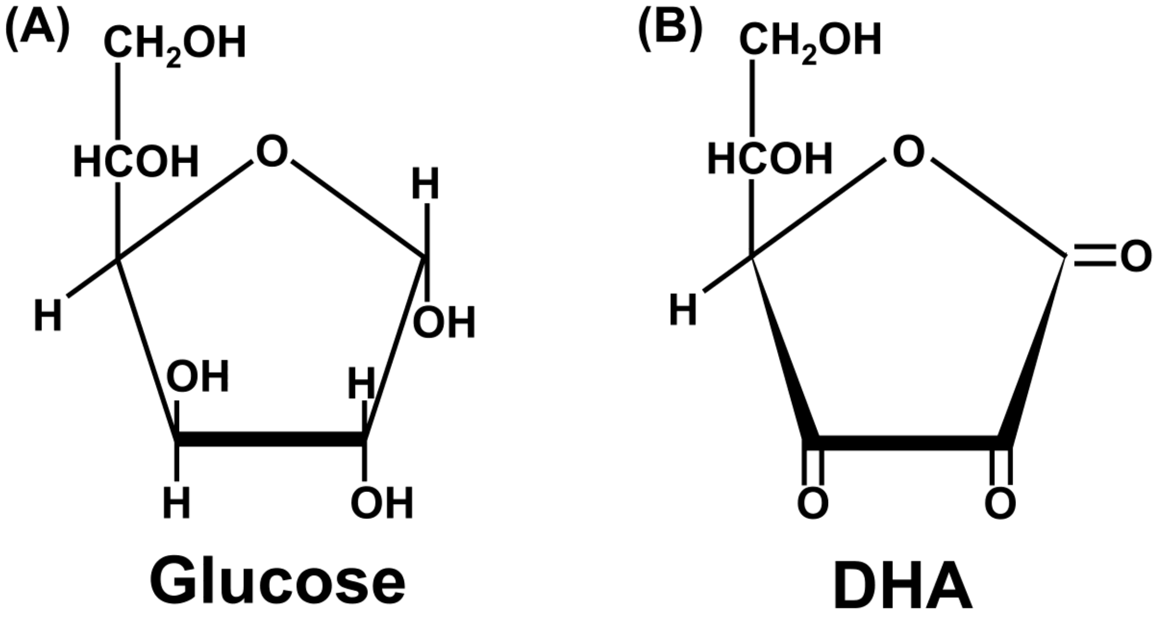

Figure S17. The chemical structural formula. (A) Glucose and (B) DHA.

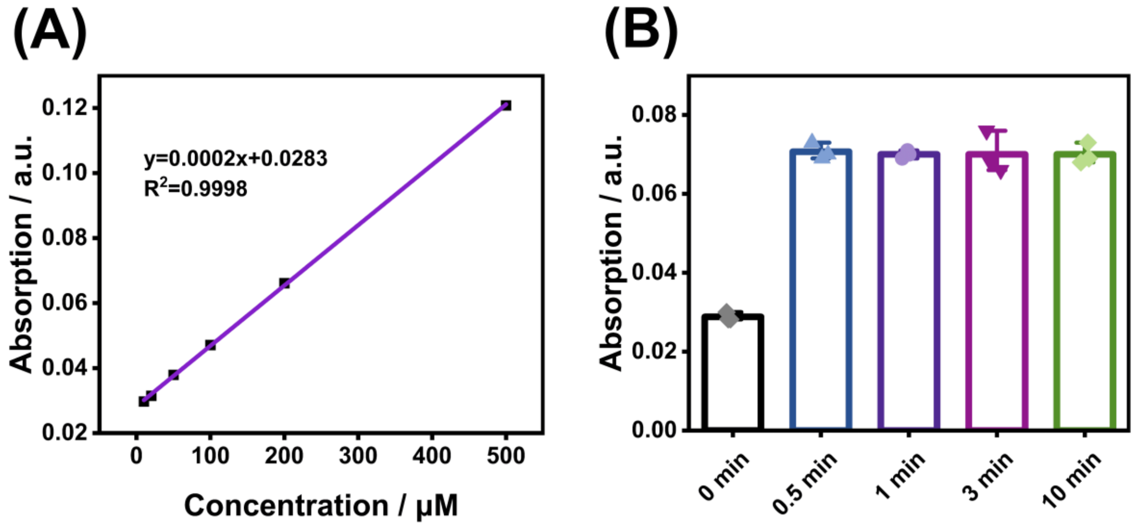

Figure S18. Verification of results of the sensor by hydrogen peroxide assay kit. (A) The calibration curve of the hydrogen peroxide assay kit measured at a wavelength of 560 nm and (B) the absorbance of samples collected at the time point before stimulation and at different time points after stimulation. (n=3)

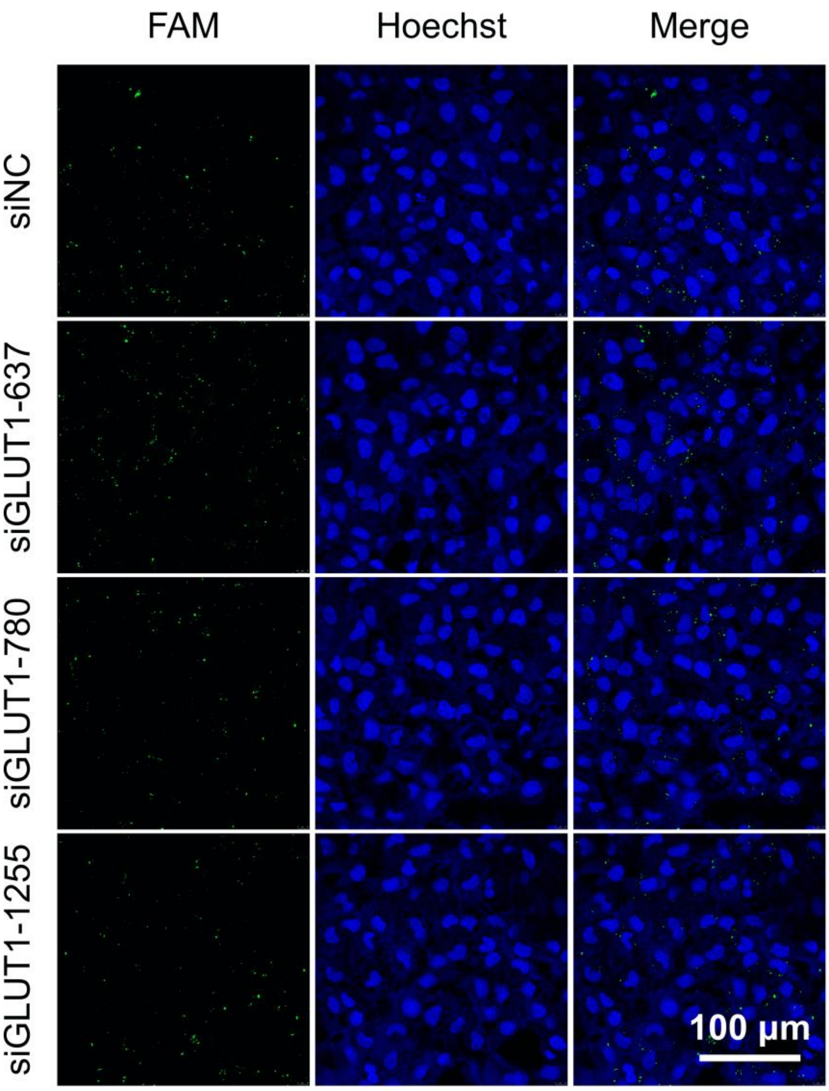

Figure S19. Record the process of fluorescence (FAM) labeled siRNA transfection into HeLa cells. Confocal immunofluorescence images of the process of fluorescence (FAM) labeled siRNA transfection into HeLa cells under different treatment conditions, including siNC (negative control), siGLUT1-637, siGLUT1-780, and siGLUT1-1255. Green: fluorescence (FAM) labeled siRNA, Blue: nucleus. Scale bar=100 μm.

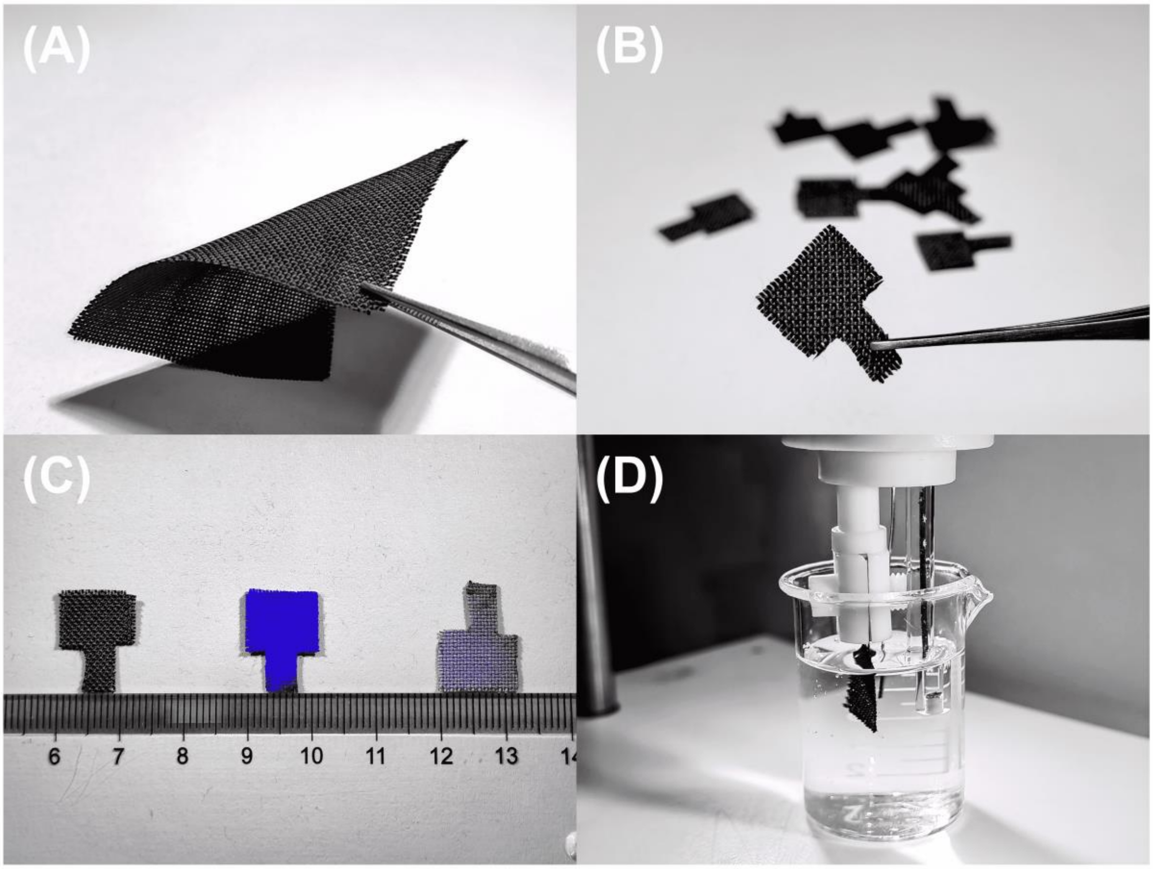

Figure S20. Photographs of the construction process of detection platform. The images of the proposed electrodes with flexible, easy to cut, and manipulate into different shapes to meet the different requirements of the experiment and the color change during electrode modification process. (A) Photographs of carbon cloth (CC) with excellent flexibility, (B) shape of CC electrode, (C) effective area of the electrode with a size of 1x1 cm^2 and the photographs of prepared electrodes of CC, Co-MOF@CC and PtNFs/CoPi@CC (from left to right). (D) The detection device with a conventional three-electrode system.

Supplemental Table

Table S1. Comparison of a proposed catalyst with reported materials for the H_2O_2 sensors.

Materials	Linear Range	LOD	Sensitivity ($\mu A/mM^{-1}cm^{-2}$)	Reproducibility (RSD)	Method	Ref
Fe_3O_4/3DG	0.8–334.4 µM	78 nM	274.15	2.3%	CA	[1]
BS-Cu-BHT film	0.5-200 µM	0.08 µM	257	4.7%	CA	[2]
MnS/O-SNC	1 µM-2.5 mM; 2.5-10 mM	0.08 µM	198.16	<2.03%	CA	[3]
$(Co\text{-}TCPP(Fe))_5$	0.4-50 µM	0.15 µM	35.4	4%	CA	[4]
PBEA	5-1000 µM	1.9 µM	21.8-23	7%	CPA	[5]
CuO–CoO-2.5 h	2 µM-4 mM	1.4 µM	6349	2.8%	CA	[6]
HCT@GNSs- PtNPs	0.0001-0.6 mM	0.05 µM	173	4.5%	CA	[7]
PtNi-N-rGO	0.01-0.05 µM; 0.15-8632 µM	2.9 nM	-	2.8%	CA	[8]
NiCo-DH/AuPt	10-670 µM; 670-22080 µM	0.145 µM	119.7; 68.9	2.79%	CA	[9]
Fe-hemin-MOFs	1-61 µM; 61-1311 µM	0.57 µM	-	4.4%	CA	[10]
Hemin/CNT/$Ti_3C_2T_x$	10-2220 µM	1.6 µM	382.17	2.2%	CA	[11]
PtNFs/CoPi	10 µM-26.64 mM	0.222 µM	274.65	2.28%	CA	This work

Table S2. Summary of the siRNA sequences used in the study.

Name	Sequences (from 5' to 3')	
Negative control	Sense	UUC UCC GAA CGU GUC ACG UTT
	Antisense	ACG UGA CAC GUU CGG AGA ATT
siGLUT1-1255	Sense	GUG CCA UAC UCA UGA CCA UTT
	Antisense	AUG GUC AUG AGU AUG GCA CTT
siGLUT1-637	Sense	CCA UGU AUG UGG GUG AAG UTT
	Antisense	ACU UCA CCC ACA UAC AUG GTT
siGLUT1-780	Sense	CUG CUG AGC AUC AUC UUC ATT
	Antisense	UGA AGA UGA UGC UCA GCA GTT

Supplemental Note

Note S1. The synthesis mechanism of Co-MOF and cobalt phosphate (CoPi).

Immersing the pretreated carbon cloth into a mixed aqueous solution of $Co(NO_3)_2{\cdot}6H_2O$ (0.05 M) and 2-methylimidazole (0.4 M) for 4 h at room temperature without stirring. (Co^{2+}: 2-methylimidazole = 1: 8).[12]

Co^{2+}+(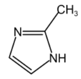 $)_2$ ↔ (Co-MOF, ZIF) (1)

A solubility equilibrium exists in the aqueous solution:

$Co^{2+}+2NO_3^-+4(N\text{-}NH) \leftrightarrow Co(N\text{-}N)_2+2(NH\text{-}NH)^+2NO_3^-$ (2)

To guarantee the formation of $Co(N\text{-}N)_2$ (Co-MOF), the molar ratio of Co^{2+}: 2-methylimidazole should be less than 1: 4. The ratio of Co^{2+}: 2-methylimidazole is 1: 8 in this work, the H^+ produced during the formation process of Co-MOF is therefore consumed by the excessive 2-methilymidazole in the solution.

$Co^{2+} + 2(N\text{-}NH) \leftrightarrow Co(N\text{-}N)_2 + 2H^+$ (3)

$(N\text{-}NH)+NO_3^-+H^+ \leftrightarrow (NH\text{-}NH) +NO_3^-$ (4)

These chemical equations below are the possible formation mechanism of Co-MOF derived PtNFs/CoPi composite based on ion exchange reaction according to the previous research.[12-14] The H^+ in hydrogen phosphate salts solution are consumed by the deprotonated 2-methlymidazole(2-MIM$^-$). Therefore, the generated PO_4^{3-}combine with Co^{2+} to form cobalt phosphate (CoPi).

$3Co(N\text{-}N)_2+2H_2PO_4^-+2HPO_4^{2-} \leftrightarrow Co_3(PO_4)_2+2PO_4^{3-}+6(N\text{-}NH)$ (5)

$Co(N\text{-}N)_2+2H_2PO_4^- \leftrightarrow Co^{2+}+2(N\text{-}NH)+2HPO_4^{2-}$ (6)

$Co(N\text{-}N)_2+2HPO_4^{2-} \leftrightarrow Co^{2+}+2(N\text{-}NH)+2PO_4^{3-}$ (7)

$3Co^{2+}+2PO_4^{3-} \leftrightarrow Co_3(PO_4)_2$ (8)

The chemical equation below is the total reaction chemical equation of the CoPi synthesis.

$15Co(N\text{-}N)_2+12H_2PO_4^-+6HPO_4^{2-} \leftrightarrow 8PO_4^{3-}+5Co_3(PO_4)_2+30(N\text{-}NH)$ (9)

Ion strength is a measure of ion concentration in the solution and a function of the concentration of all ions in the solution. It is defined as follows:

$$I_i = \frac{1}{2}\sum_{i=1}^{n} C_i Z_i^2.$$

C_i is the mass molar concentration of ion i, and Z_i is the number of charges carried by the ion i. From the perspective of ion strength, during the ion exchange reaction process, we assume that the concentration of $H_2PO_4^-$ is c_1 ($c(H_2PO_4^-)=c_1$), similarly, $c(HPO_4^{2-})=c_2$, $c(PO_4^{3-})=c_3$. After obtaining the proton, deprotonated 2-MIM (2-MIM$^-$) becomes 2-MIM (a simple neutral

organic compound) which has no contribution to ion strength. At the same time, since Na^+ do not participate in any reactions, their concentration is always a constant in the formula for calculating ion strength.

Ion strength (I_i)= $\frac{1}{2}\times c(Co^{2+})\times 4+\frac{1}{2}\times c(Na^+)\times 1+\frac{1}{2}\times(c_1)\times 1+\frac{1}{2}\times(c_2)\times 4+\frac{1}{2}\times(c_3)\times 9$, the concentration of Co^{2+} increases from zero to a stable value, $c(Na^+)$ is a constant, as the reaction progresses, c_1 decreases, c_2 decreases while c_3 increases. Due to its large coefficient of phosphate radical, it is the main factor affecting ion strength. For example, assuming a reaction of one mole occurs, the $\frac{1}{2}\times(c_1)\times 1+\frac{1}{2}\times(c_2)\times 4$ reduced 18 ($\frac{1}{2}\times 12\times 1+\frac{1}{2}\times 6\times 4=18$), the $\frac{1}{2}\times(c_3)\times 9$ increased 36 ($\frac{1}{2}\times 8\times 9=36$), therefore, the ion strength increases more than 18 (36-18=18) after adding the contribution of cobalt ions to the calculation of ion strength. All in all, the ion strength increases as the ion exchange reaction progresses.

Note S2. The corresponding chemical structural formula.

$(N-N)^-$ represents the deprotonated 2-methlymidazole (2-MIM$^-$): 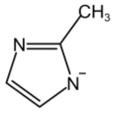

(N-NH) represents the 2-methlymidazole (2-MIM): 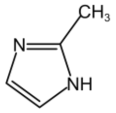

$(NH-NH)^+$ represents the pronated 2-methlymidazole (2-MIMH$^+$): 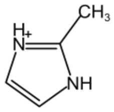

Supplementary References

[1] Y. Zhao, D. Huo, J. Bao, M. Yang, M. Chen, J. Hou, H. Fa, C. Hou, *Sens. Actuator B-Chem.* **2017**, 244, 1037.

[2] X. Chen, J. Dong, K. Chi, L. Wang, F. Xiao, S. Wang, Y. Zhao, Y. Liu, *Adv. Funct. Mater.* **2021**, 31, 2102855.

[3] K. Shim, K. D. Seo, H. J. Kim, *Adv. Funct. Mater.* **2023**, 2210549.

[4] Y. Wang, M. Zhao, J. Ping, B. Chen, X. Cao, Y. Huang, C. Tan, Q. Ma, S. Wu, Y. Yu, Q. Lu, J. Chen, W. Zhao, Y. Ying, H. Zhang, *Adv. Mater.* **2016**, 28, 4149.

[5] D. Rojas, J. F. Hernandez-Rodriguez, F. Della Pelle, M. Del Carlo, D. Compagnone, A. Escarpa, *Biosens. Bioelectron.* **2020**, 170, 112669.

[6] W. Zhang, G. Fan, H. Yi, G. Jia, Z. Li, C. Yuan, Y. Bai, D. Fu, *Small* **2018**, 14, 1703713.

[7] Y. Zhang, K. Chi, J. Xiao, Y. Xu, A. Zhao, Y. Xu, Y. Sun, F. Xiao, S. Wang, *Biosens. Bioelectron.* **2020**, 150, 111924.

[8] Y. Yu, J. Peng, M. Pan, Y. Ming, Y. Li, L. Yuan, Q. Liu, R. Han, Y. Hao, Y. Yang, D. Hu, H. Li, Z. Qian, *Small Methods* **2021**, 5, 2001212.

[9] J. Zhao, H. Yang, W. Wu, Z. Shui, J. Dong, L. Wen, X. Wang, M. Yang, C. Hou, D. Huo, *Anal. Chim. Acta* **2021**, 1143, 135.

[10] P. Zhao, S. Chen, J. Zhou, S. Zhang, D. Huo, C. Hou, *Anal. Chim. Acta* **2020**, 1128, 90.

[11] P. Zhao, S. Chen, Y. Liang, Y. Chen, P. Lan, D. Huo, C. Hou, *J. Colloid Interface Sci.* **2022**, 628, 456.

[12] B. Zhang, Z. Qi, Z. Wu, Y. H. Lui, T.-H. Kim, X. Tang, L. Zhou, W. Huang, S. Hu, *ACS Energy Lett.* **2018**, 4, 328.

[13] Y. Zhang, H. Chen, C. Guan, Y. Wu, C. Yang, Z. Shen, Q. Zou, *ACS Appl. Mater. Interfaces* **2018**, 10, 18440.

[14] H. Chen, Z. Shen, Z. Pan, Z. Kou, X. Liu, H. Zhang, Q. Gu, C. Guan, J. Wang, *Adv. Sci.* **2019**, 6, 1802002.
